# Changes in the Fraction Metabolized in Children Younger than Four Years; When is Clearance Scaling for the Dominant Elimination Route in Adults Appropriate?

**DOI:** 10.1007/s11095-025-03948-7

**Published:** 2025-10-14

**Authors:** Anne van Rongen, Robbin Grijseels, Elisa A. M. Calvier, Karel Allegaert, Catherijne A. J. Knibbe, Elke H. J. Krekels

**Affiliations:** 1 Division of Systems Pharmacology and Pharmacy, Leiden Academic Center for Drug Research, Leiden University, Einsteinweg 55, 2333 CC, Leiden, Netherlands; 2 Pharmacokinetics-Dynamics and Metabolism, Translational Medicine and Early Development, Sanofi R&D, Montpellier, France; 3 Department of Development and Regeneration, KU Leuven, Leuven, Belgium; 4 Department of Pharmaceutical and Pharmacological Sciences, KU Leuven, Leuven, Belgium; 5 Department of Hospital Pharmacy, Erasmus MC, Rotterdam, Netherlands; 6 Department of Clinical Pharmacy, St. Antonius Hospital, Nieuwegein and Utrecht, The Netherlands; 7 Certara Inc, Princeton, NJ USA

**Keywords:** CYP, maturation, physiologically-based pharmacokinetics, SULT, UGT

## Abstract

**Introduction:**

A common approach to scaling clearance from adults to children is to apply a maturation function for the dominant elimination pathway in adults. We investigate for drugs mainly cleared through hepatic metabolism, how the fraction metabolized changes and whether this pathway remains dominant in young children.

**Methods:**

A physiologically-based pharmacokinetic workflow was developed investigating 460 hypothetical drugs that were for 90% or 70% cleared through hepatic metabolism in adults with remaining clearance through glomerular filtration. Their unbound drug fractions were between 1 and 99% and they were metabolized by isoenzymes with different maturation patterns. Absolute and relative clearance through hepatic metabolism was calculated in a typical adult and seven typical pediatric individuals younger than 4 years.

**Results:**

When hepatic metabolism comprises 90% of total plasma clearance in adults, it tends to remain the dominant elimination route throughout childhood for substrates of all isoenzymes, except for substrates of CYP2A6, UGT1A1, and UGT2B7. However, when hepatic metabolism comprises 70% of the total plasma clearance in adults, hepatic metabolism will not remain dominant for at least part of the pediatric age-range for substrates of many enzymes, except for substrates of CYP2C8, CYP2C9, and SULT1A1.

**Conclusion:**

This study identified scenarios in which hepatic metabolism cannot be assumed to remain dominant in children younger than 4 years, when it is the dominant elimination route in adults. In these scenarios, scaling for the dominant clearance route in adults will yield underprediction of total plasma clearance and the contribution of alternative routes needs to be considered.

**Supplementary Information:**

The online version contains supplementary material available at 10.1007/s11095-025-03948-7.

## Introduction

**Fig. 1:**
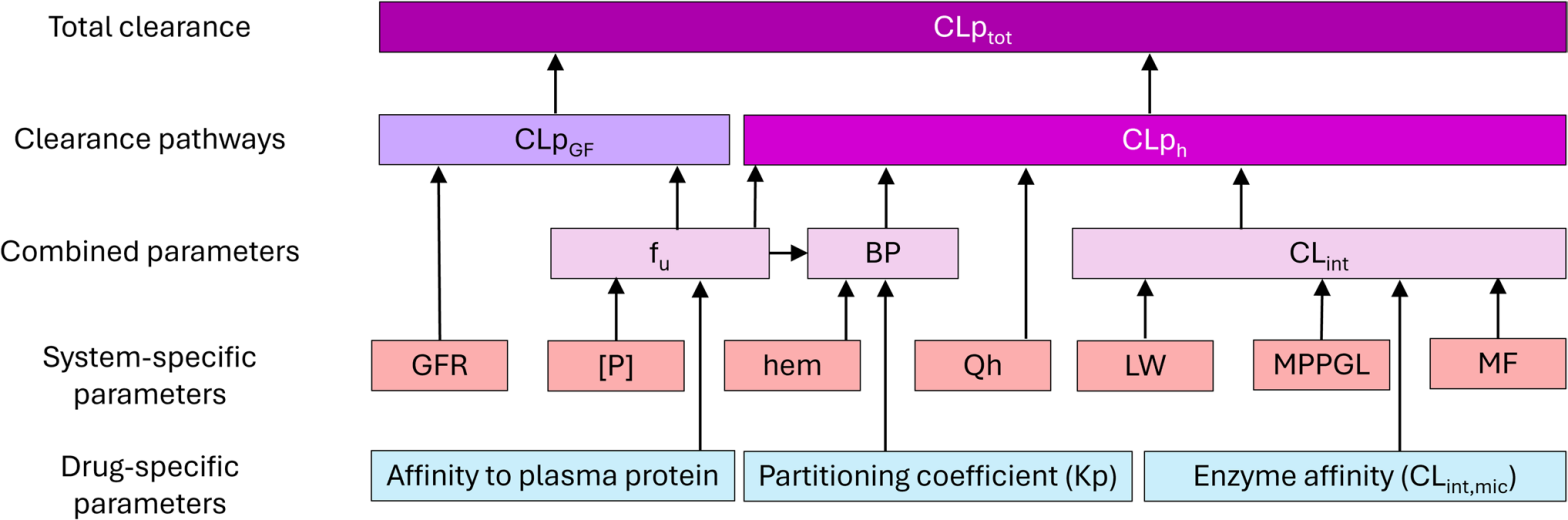
Overview of how drug-specific and system-specific parameters are related to combined parameters, plasma clearance through glomerular filtration (CLp_GF_), plasma clearance through hepatic metabolism (CLp_h_), and total plasma clearance (CLp_tot_). BP = blood-to-plasma ratio; CL_int_ = whole-liver intrinsic clearance; f_u_ = unbound drug fraction; GFR = glomerular filtration rate; hem = hematocrit; LW = liver weight; MF = enzyme maturation factor; MPPGL = microsomal protein per gram of liver; [P] = concentration of the plasma protein to which a drug binds; Qh = hepatic blood flow.

Multiple variables drive total plasma clearance as illustrated in Fig. 1. At birth, most system-specific parameters are immature with maturation rates tending to be non-linear and different for each parameter. The impact of each of these changes on combined parameters (i.e., unbound drug fraction (f_u_), blood-to-plasma ratio (BP), and whole liver intrinsic clearance (CL_int_)), on individual clearance routes (i.e., hepatic metabolism or glomerular filtration), and ultimately on total plasma clearance (CLp_tot_), depends on the properties of the specific drug (i.e., affinity to plasma proteins, the partitioning coefficient (Kp), and enzyme affinity which drives intrinsic microsomal clearance (CL_int,mic_). With total plasma clearance representing the sum of all renal and hepatic clearance pathways, it varies throughout childhood, with a specific maturation pattern for each drug.

As total plasma clearance is one of the main determinants for maintenance drug doses, describing maturation of drug clearance across the pediatric age-range is imperative for defining drug dosing algorithms for the pediatric population. In the absence of clinical data on a drug, for instance during drug development or for off-label pediatric dosing, commonly applied approaches to predicting pediatric plasma clearance may take the dominant clearance route in adults and scale drug clearance to the pediatric population based on a maturation function for the dominant clearance pathway, or may scale clearance based on bodyweight followed by inclusion of additional corrections based on the dominant clearance pathway. These approaches, however, do not consider that minor clearance pathways in adults may take over during childhood when the major adult pathway is still immature. The main clearance pathway for paracetamol and propofol in adults is, for instance, glucuronidation, while this is sulfation [1] and hydroxylation [2], respectively, in newborns and infants. Similarly, caffeine is mainly metabolized in adults, whereas renal excretion dominates in the first month of life [3]. Not accounting for minor pathways that take over drug elimination during childhood could lead to an underprediction of pediatric clearance and pediatric dose requirements.

Here, we explore scenarios for drugs that are mainly cleared through hepatic metabolism in adults, to establish to what extent this pathway can be assumed to remain dominant in young children. For this, a physiologically-based pharmacokinetic (PBPK) modelling framework is used to investigate for drugs with a wide range of drug-properties how the fraction metabolized changes in children of 4 years and younger.

## Materials and Methods

A PBPK-based simulation framework was developed in R (version 4.4.3) analogue to previous publications by Calvier *et al.* [4, 5]. PBPK-models provide a comprehensive and well-established framework to integrate existing pharmacological knowledge to predict pharmacokinetic parameters based on drug-specific and system-specific variables [6–12]. The current framework included a PBPK model for drug clearance through hepatic metabolism and glomerular filtration.

Simulations were performed with this model for 460 hypothetical drugs with different drug properties in a virtual population of a typical adult and typical pediatric individuals with an age of 1 day, 2 weeks, 1 month, 6 months, and 2, 3, or 4 years. The upper age limit was selected as most isoenzymes mature within the first 4 years of life. Given that most drugs are cleared at least to some degree through glomerular filtration and given that GFR already has a considerable capacity at birth, this route was selected as the minor adult pathway in all scenarios.

To obtain insights into the impact of isoenzyme maturation on the fraction metabolized, one set of simulations was performed in which the studied drugs were assumed to be substrates for hypothetical isoenzymes that had a constant degree of enzyme maturation in the first 4 years of life. Subsequently, drugs with the same properties were investigated that were substrates for existing isoenzymes to investigate changes in the fraction metabolized resulting from isoenzyme-specific maturation patterns.

### Physiologically-based Pharmacokinetic Model for Multiple Pathways

Hepatic metabolic plasma clearance (CLp_h_) was calculated using the dispersion model, which is based on unbound drug fraction (f_u_), whole-liver intrinsic clearance (CL_int_), blood-to-plasma ratio (BP), and hepatic blood flow (Qh), according to Eqs. 1–6:

1 $${CLp}_{h}={CL}_{B}\cdot BP$$

2 $${CL}_{B}={Q}_{H}\cdot ER$$

3 $$ER=1-{F}_{H}$$

4 $${F}_{H}=\frac{4a}{{(1+a)}^{2}\cdot \text{exp}\left\{\left(a-1\right)/2\cdot {D}_{N}\right)\}-{\left(1-a\right)}^{2}\cdot \text{exp}\{-(a+1)/2\cdot {D}_{N}\}}$$

5 $$a={(1+4\cdot {R}_{N}\cdot {D}_{N})}^{1/2}$$

6 $${R}_{N}={f}_{u}/BP\cdot {CL}_{int}/{Q}_{H}$$

In these equations, *CL*_*B*_ is the whole-blood clearance, *ER* is the hepatic extraction ratio, *F*_*H*_ is the fraction escaping hepatic metabolism (hepatic bioavailability), *D*_*N*_ is the axial dispersion number for which 0.71 was used [13], and *R*_*N*_ is the efficiency number.

Plasma clearance through glomerular filtration (CLp_gf_) was quantified as the product of GFR and unbound drug fraction (f_u_), as described in Eq. 7:

7 $${CLp}_{gf}=GFR\cdot {f}_{u}$$

### Hypothetical Drugs

Twenty drugs were generated. Each drug was assumed to exclusively bind to either human serum albumin (HSA) or α1-acid glycoprotein (AGP) and had an affinity for these plasma proteins that led to an f_u_ in the typical adult of 1%, 25%, 50%, 75%, or 99%. For each drug, the intrinsic microsomal clearance (CL_int,mic_) was selected to be such that clearance in the typical adult comprised of 90% or 70% hepatic metabolism, with the remaining elimination route being glomerular filtration. This meant that the CL_int,mic_ values were 0.0162, 0.0188, 0.0216, 0.0246, and 0.0278 mL/min per mg of microsomal protein, for drugs with an f_u_ of 1%, 25%, 50%, 75%, or 99%, respectively and 90% hepatic metabolism in adults and 0.00417, 0.00432, 0.00445, 0.00455, and 0.00464 mL/min per mg of microsomal protein, for drugs with the same f_u_ and 70% hepatic metabolism in adults. The partitioning coefficient (Kp), which drives the blood-to-plasma ratio (BP), was set to 1 for all drugs, meaning that all drugs distribute equally into plasma and red blood cells.

Each of the 20 drugs described above were simulated to be substrates for one of seven hypothetical isoenzymes with a constant state of maturation of 100%, 75%, 50%, 25%, 10%, 5%, or 0.5% in all seven pediatric individuals (e.g., a flat function was used to describe the maturation of these hypothetical isoenzymes in the studied pediatric subjects, while the value was 100% in the typical adult). This does not reflect a clinical scenario, but was included to assess the impact of isoenzyme maturation in conjunction with maturational changes in the other physiological processes driving drug clearance. In addition, each drug was also simulated to be a substrate of one of the following sixteen isoenzymes that are commonly involved in drug metabolism: CYP1A2, CYP2A6, CYP2B6, CYP2C8, CYP2C9, CYP2C18, CYP2C19, CYP2D6, CYP2E1, CYP3A4, UGT1A1, UGT1A4, UGT 1A6, UGT1A9, UGT2B7, or SULT1A1. Twenty compounds being substrate of one of 7 hypothetical or 16 real isoenzymes, yields a total of 460 investigated drugs.

### Virtual Population

Simulations were performed for a typical adult of 25 years and for seven typical pediatric individuals of 1 day, 2 weeks, 1 month, 6 months, and 2, 3, or 4 years. All individuals were assumed to be born at term (i.e., with a gestational age of 38 weeks). Typical weights and heights were obtained from CDC growth charts [14] and calculated as the average value for males and females. Body surface area (BSA) was calculated according to Haycock *et al.* [15] for typical children ≤ 15 kg and according to Du Bois and Du Bois [16] for individuals > 15 kg. Equations to derive pediatric values for the system-specific parameters were taken from Johnson *et al.* [8], unless stated otherwise.

Maturational changes in unbound drug fraction (f_u_) were assumed to be solely driven by changes in the concentration of drug binding plasma protein [P], according to Eq. 8 [8]:

8 $${f}_{u,pediatric}=\frac{1}{1+\frac{(1-{f}_{u,adult})\cdot {[P]}_{pediatric}}{{[P]}_{adult}\cdot {f}_{u,adult}}}$$

Concentration of HSA and AGP were calculated for each typical individual using Eqs. 9 and 10, respectively [8]:

9 $$HSA=1.1287\cdot \text{ln}\left(Age\right)+33.746$$

10 $$AGP=\frac{0.887\cdot {Age}^{0.38}}{{8.89}^{0.38}+{Age}^{0.38}}$$

Both equations express protein concentrations as g/L and use *Age* in days.

The whole-liver intrinsic clearance (CL_int_) for each drug in each typical individual was derived from liver weight (LW), microsomal protein per gram of liver (MPPGL), an age specific and isoenzyme specific maturation factor (MF), and the intrinsic clearance per mg of microsomal protein (CL_int,mic_) according to Eq. 11 [8]:

11 $${CL}_{int}=LW\cdot MPPGL\cdot MF\cdot {CL}_{int,mic}$$

In this equation, *LW* in g was obtained by multiplying the liver density, which was assumed to be 1080 g/L and age-independent, with the liver volume (LV) in L being derived from Eq. 12 [8]:

12 $$LV=0.722\cdot {BSA}^{1.176}$$

*CL*_*int,mic*_ was age-independent and calculated for each hypothetical drug to yield 90% or 70% clearance through hepatic metabolism in the typical adult, as mentioned previously. Age-dependent values for *MPPGL* were obtained from the Simcyp® V15.R1 library. The maturation factor (MF) for each of the “real” hepatic isoenzymes were obtained from the Simcyp® V15.R1 library except for SULT1A1, which was taken from Hines [17]. Supplemental Fig. 1 and Table 1 in Supplement 1 illustrate for each isoenzyme included in the analysis, how the maturation factor (MF) changes with age.

The partitioning coefficient (Kp) was assumed to be age-independent and remained constant at a value of 1, as mentioned previously. As a result, the blood-to-plasma ratio (BP) was assumed to only change due to changes in unbound drug fraction (f_u_) and hematocrit (hem), according to Eq. 13 from Maharaj *et al.* [18]:

13 $$BP=1+hem\cdot ({f}_{u}\cdot {K}_{p}-1)$$

The values of *hem* for each typical individual were taken from the Simcyp® V15.R1 library.

Hepatic blood flow (Qh) was assumed to be 27.5% of the cardiac output (CO) at all ages. CO was set to be 6.2 L/min for the typical adult, which is the average value of male and female adults in the ICRP publication 89 [19], while Eq. 14 was used to calculate CO in the typical pediatric individuals [8]:

14 $$CO=BSA\cdot (2.5+\frac{\left(Age-1\right)\cdot \left(4\cdot 2.5\right)}{\left(10-1\right)})$$

In Eq. 14, *Age* is expressed in years and it should be noted that this equation is only valid in children of 10 years and younger.

For age-specific GFR values, Eq. 15, obtained by Rhodin *et al.* [20], was used:

15 $$GFR=112\cdot\left(\frac{WT}{70}\right)^{0.635}\cdot\left(\frac{{PMA}^{3.33}}{{55.4}^{3.33}+{PMA}^{3.33}}\right)$$

This equation is based on bodyweight (WT) in kg and post-menstrual age (PMA) in weeks.

Supplemental Table 1 in supplement 1, provides an overview of all variables and parameter values used in the simulations for the typical individuals in the virtual population.

### Simulated Scenarios

Hepatic metabolic plasma clearance (CLp_h_) and plasma clearance through glomerular filtration (CLp_gf_) were simulated for all hypothetical drugs in each typical individual from the virtual population. Total plasma clearance (CLp_tot_) was defined as the sum of these two clearance routes. From this value, it was calculated what percentage of the total drug clearance each route comprised, with the contribution of CLp_h_ representing the fraction metabolized.

## Results

**Fig. 2:**
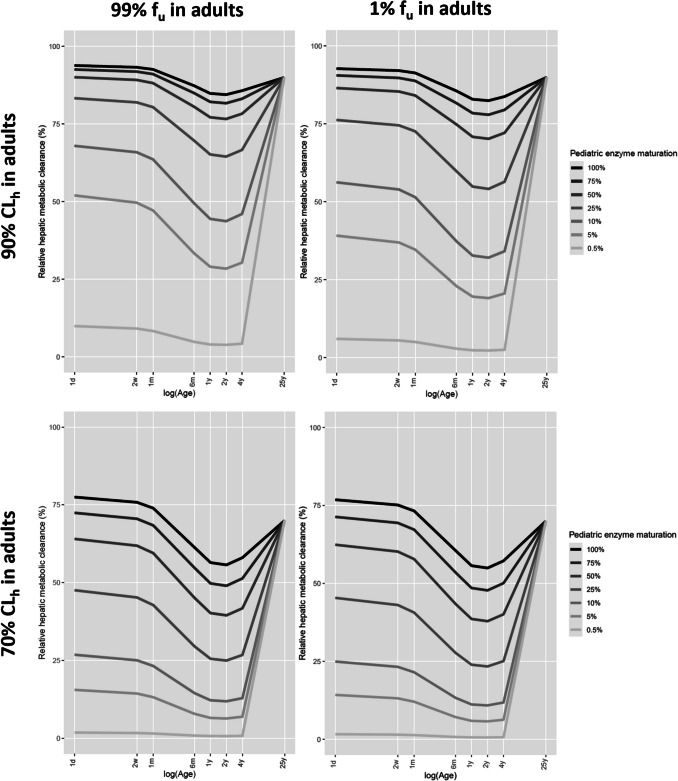
Relative contribution of hepatic metabolic plasma clearance to total plasma drug clearance (i.e., percentage metabolized) for drugs binding to human serum albumin with an unbound drug fraction (f_u_) in adults of 99% (left) or 1% (right) that are substrates for hypothetical isoenzymes that have a constant degree of pediatric enzyme maturation (i.e., the same maturation factor (MF) for all seven pediatric ages) as indicated in the legend. The scenarios reflect drugs with an intrinsic microsomal clearance (CL_int,mic_) that yields total plasma clearance in the typical adult to be comprised of 90% (top) or 70% (bottom) hepatic metabolism with the remaining clearance being through glomerular filtration.

Figure 2 shows the relative contribution of hepatic metabolic plasma clearance to total plasma clearance for various hypothetical drugs binding to HSA with an unbound drug fraction (f_u_) in adults of 99% or 1% that are substrates for hypothetical enzymes that have a constant maturation factor throughout the pediatric population (i.e., the same MF for all seven pediatric ages). The remaining fraction that is cleared through glomerular filtration in all scenarios, is not shown but is represented by 1 minus the fraction cleared through hepatic metabolism. Additional graphs can be found in Supplement 2 for drugs being cleared for 90% through hepatic metabolism in adults and in Supplement 3 for drugs being cleared for 70% through hepatic metabolism in adults. These graphs show that the relative contribution of hepatic metabolism for drugs with unbound drug fractions (f_u_) values between 99 and 1% lie in between the values in the presented plots and that results for drugs binding to AGP are very similar to the results for drugs binding to HSA.

It can be seen in Fig. 2, Supplement 2, and Supplement 3, that for a drug that is substrate for an isoenzyme that is mature at birth (i.e., MF is at 100% at all pediatric ages), maturational changes in all other system-specific parameters (i.e., glomerular filtration rate (GFR), hepatic blood flow (Qh), hematocrit (hem), liver weight (LW), and microsomal protein per gram of liver (MPPGL)) result in the contribution of hepatic metabolism slightly exceeding the adult values (i.e., 90% and 70%) in early life, with a 10% – 20% decrease in contribution of hepatic metabolism between the ages of 6 months and 4 years. The contribution of hepatic metabolic clearance to total plasma clearance is decreased more for drugs with higher protein binding (i.e., lower f_u_ in adults), with this effect being more pronounced for drugs with a higher percentage of clearance through hepatic metabolism in adults. This suggests that maturational changes in unbound drug fraction (f_u_), resulting from age-related changes in protein concentrations ([P]), have a bigger impact on hepatic metabolic plasma clearance than on plasma clearance through glomerular filtration.

When hepatic metabolism in adults comprises 90% of total plasma clearance, hepatic metabolism remains the dominant elimination pathway throughout childhood, even when the enzyme maturation factor (MF) drops to 25% of adult values. At 10% MF, glomerular filtration becomes the dominant elimination route in children between 6 months and 4 years, but in children of 1 month and younger hepatic metabolism is still dominant. Only at 5% enzyme maturation or lower does GFR become the dominant elimination route for all drugs in all ages below 4 years. When hepatic metabolism in adults comprises 70% of total plasma clearance, however, glomerular filtration is already dominant between the ages of 1–4 years, when MF is at or below 75%, while at an MF of 25% glomerular filtration is dominant in all ages below 4 years.

**Fig. 3:**
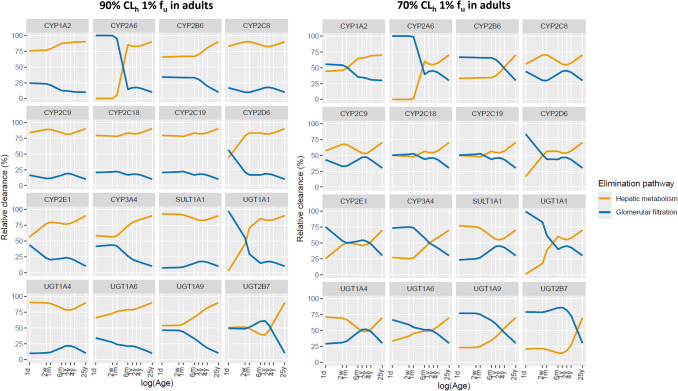
Relative contribution of hepatic metabolism (i.e., percentage metabolized) and glomerular filtration in the typical adult and pediatric individuals for drugs binding to human serum albumin with an unbound drug fraction (f_u_) in adults of 1% that are substrates for the indicated isoenzymes and that are for 90% (left) or 70% (right) metabolized through hepatic metabolism in adults.

Figure 3 illustrates how the contribution of hepatic metabolic clearance and glomerular filtration changes with age for the drugs that are substrates for 16 isoenzymes commonly involved in drug metabolism. The figure shows results for drugs binding to HSA that have an unbound drug fraction (f_u_) in adults of 1% and that are for 90% or 70% cleared through hepatic metabolism in adults. Remaining plots for drugs binding to HSA or AGP with different f_u_ values in adults can be found in Supplement 4 for drugs that are for 90% cleared through hepatic metabolism in adults and in Supplement 5 for drugs that are cleared for 70% through hepatic metabolism.

Based on published enzyme maturation profiles in Supplement 1, hepatic metabolism will remain the dominant pathway throughout childhood for substrates of most isoenzymes for drugs for which the contribution of hepatic metabolic clearance to total plasma clearance is 90% in adults. This is irrespective of the degree of protein binding or the plasma protein the drug binds to. For substates of UGT2B7, the contribution of hepatic metabolism and glomerular filtration fluctuate around 50% in children of 4 years and younger, with GFR tending to be more dominant between the ages of 6 months and 4 years and for drugs with a lower unbound drug fraction in adults. For drugs that are substrate for CYP2A6 and UGT1A1, GFR will almost completely take over as clearance pathway in children below the age of 6 months or at the age of 2 weeks and younger, respectively. For drugs that are for 70% cleared through hepatic metabolism in adults, hepatic metabolism will only remain dominant for substrates of CYP2C8, CYP2C9, and SULT1A1. For substrates of the remaining isoenzymes, the contribution of hepatic metabolism generally remains above 25% throughout childhood, except for CYP2A6 and UGT1A1 for which GFR will almost completely take over as clearance pathway in children below the age of 6 months or 1 month and younger, respectively. Table I provides an overview of which clearance pathway is dominant for substrates of the isoenzymes. As long as details on isoenzyme maturation are known, the results obtained for substrates of the hypothetical isoenzymes can be used to make inferences about changes in fraction metabolized of substrates for real isoenzymes that have not be included in the current analysis. In these cases Fig. 2 can be used to determine for each age and associated fraction of enzyme activity (MF), what the fraction metabolized in children will be.

**Table I.**
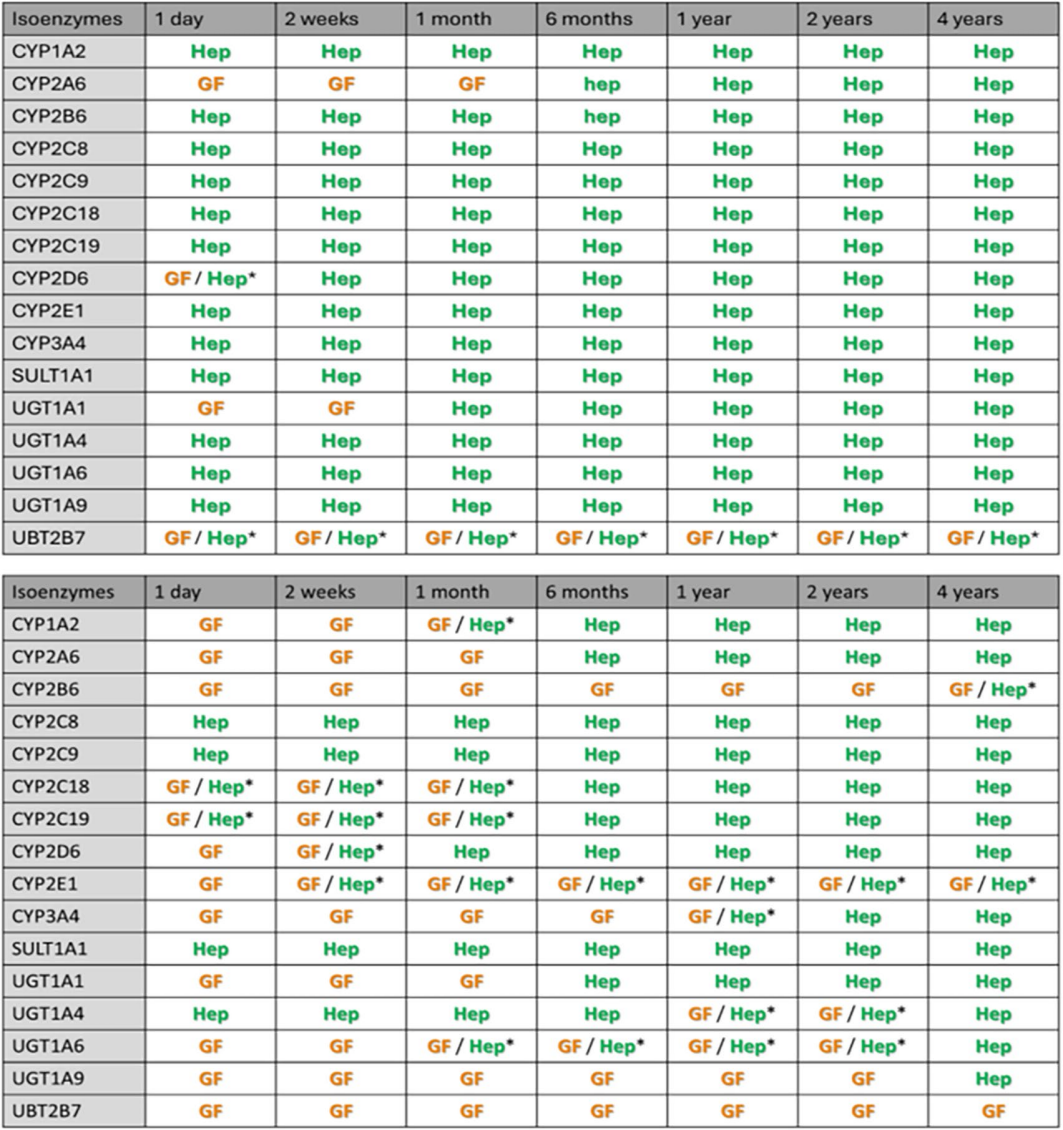
Overview of Whether Hepatic Metabolism (HEP) or Glomerular Filtration (GF) is the Major Elimination Pathway in Children of Various Ages for Drugs That in Adults Are for 90% (top) or 70% (bottom) Cleared Through Hepatic Metabolism By the Indicated Isoenzyme and With the Remaining Clearance Being Through Glomerular Filtration

^*^Clearance through hepatic metabolism and glomerular filtration fluctuates around 50% with GF tending to be more dominant when the unbound drug fraction (f_u_) in adults is lower

The plots of changes in relative clearance pathway contributions in Fig. 2 and 3 do not explicitly reflect that absolute values for hepatic metabolic plasma clearance (CLp_h_), plasma clearance through glomerular filtration (CLp_gf_), and total plasma clearance (CLp_tot_) decrease in the pediatric individuals compared to adult values. Horizontal patterns in relative contribution of hepatic metabolic plasma clearance and plasma clearance through glomerular filtration with age, indicate that both clearance through hepatic metabolism and clearance through glomerular filtration decrease with the same fraction. How the absolute clearance values change for each hypothetical drug can be found in Supplement 4 and 5.

## Discussion

This study uses a PBPK-based workflow to investigate for drugs that are predominantly cleared through hepatic metabolism in adults with a minor contribution of glomerular filtration, how the fraction metabolized changes in young children and what implications this has for the application of clearance scaling approaches based on the most dominant clearance route in adults. The advantage of the applied PBPK-model-based approach is that it yields “clean” clearance values for specific elimination routes based on established physiological and pharmacological knowledge, while clearance values from real drugs may be impacted by unknown clearance routes or may be confounded by intrinsic and extrinsic patient characteristics. Moreover, as clearance cannot be directly measured, clinical values can only be derived indirectly with a certain degree of uncertainty from observations taken with measurement error and a sampling design that is likely suboptimal, particularly in pediatric subjects. Finally, by studying all possible combinations of realistic variable values, the PBPK-based framework allowed for a systematic evaluation of the entire drug-specific parameter space, where studies based on clinical data would be limited to drugs that have been hitherto developed and studied in children. As such, this study provides general insight, rather than guidance on specific drugs.

Enzyme maturation (MF) and maturational changes in unbound drug fraction (f_u_) are important drivers for changes in absolute and relative hepatic metabolic clearance. The contribution of hepatic metabolism decreases with decreasing enzyme maturation. Additionally, decreases in pediatric plasma clearance are stronger for drugs with a higher protein binding (i.e., lower unbound drug fraction (f_u_) in adults), with this effect being more pronounced for drugs with a higher percentage of clearance through hepatic metabolism in adults. Changes in other system-specific parameters (i.e., GFR, Qh, hem, LW, and MPPGL) only have a limited impact on the contribution of hepatic metabolism in children, causing a minor decrease in the relative contribution of hepatic metabolism of about 10–20% in children around 6 months to 4 years or age, relative to the younger or older ages.

The results show that when hepatic metabolism comprises 90% of the total plasma clearance in adults, it tends to remain the dominant elimination route throughout childhood for substrates of most studied isoenzymes. However, when hepatic metabolism comprises 70% of the total plasma clearance in adults, it only remains dominant for substrates of a small number of studied isoenzymes, particularly at or below 1 month of age, glomerular filtration tends to become dominant.

Reported observations for caffeine clearance are in line with our findings. Caffeine is for 70–80% metabolized by CYP1A2 in adults and to a lesser extent by CYP2E1 and CYP3A4 [21]. Our simulations show hepatic metabolism through all these enzymes to be considerably decreased in childhood, particularly in the neonatal period, which would explain the shift towards renal excretion seen for this compound in the first month of life. Propofol metabolism in adults is driven by UGT1A9 [2] and paracetamol metabolism by UGT1A6 and 1A9 [22]. For both isoenzymes the contribution of hepatic metabolism to total drug clearance in our simulations decreases in young children. In our scenarios, the only available alternative route for drug clearance was glomerular filtration, but observations for propofol and paracetamol illustrate that other, more mature, pathways can take over as dominant elimination route as well. In the case of propofol this is hydroxylation by CYP enzymes [2] and for paracetamol that is sulfation through SULT enzymes [23]. To investigate this further the developed framework can easily be extended to include different or multiple minor elimination pathways, including active tubular secretion or reabsorption, provided that maturation functions for these routes are known. The applied methodology is furthermore limited to term-born pediatric subjects, because there are currently gaps in our knowledge regarding developmental and maturational variations in system-specific variables in this population. Once functions describing development and maturation of these variables in this population become available, the framework can be extended to this population as well.

In scenarios in which hepatic metabolism remains dominant in a pediatric age-range, scaling total drug clearance in adults to pediatric drug clearance based on scaling for the dominant metabolic elimination route in adults will be appropriate. However, at ages and for drug where hepatic metabolism does not remain dominant, total pediatric drug clearance scaling based on scaling for the most dominant clearance route in adults will yield underprediction of total plasma clearance and of the pediatric dose requirements. In these cases, the contributions of alternative clearance routes need to be taken into account, using for instance physiologically-based scaling approaches, to achieve accurate clearance scaling. Additionally, it should be considered that in case of impairment in the elimination route that takes over during childhood, the clearance of a drug in children may be impacted more than one would expect based on the major elimination route in adults. For instance, for those drugs where in children renal excretion takes over as dominant elimination pathway from hepatic metabolism, the impact of renal dysfunction on the total clearance of the drug in children, may be more relevant than one may expect for a drug that is mainly cleared through hepatic metabolism in adults.

## Conclusion

In young children, the fraction of a drug that is metabolized changes as a result of differential maturation patterns of various elimination routes. When hepatic metabolism comprises 90% of total adult plasma clearance, hepatic metabolism remains the dominant route of elimination throughout childhood for substrates of most, but not all, isoenzymes. However, when hepatic metabolism in adults comprises 70% of total plasma clearance, hepatic metabolism will not remain dominant for at least part of the pediatric age-range for substrates of most, but not all, isoenzymes. In scenarios where hepatic metabolism remains dominant in the pediatric subjects, clearance scaling for the dominant clearance route in adults is appropriate. When hepatic metabolism does not remain dominant, this scaling approach will yield underprediction of total plasma clearance and the contribution of alternative routes needs to be taken into account.

## Supplementary Information

Below is the link to the electronic supplementary material.

Supplementary Material 1 (DOCX 99.8 KB)

Supplementary Material 2 (PDF 19.0 KB)

Supplementary Material 3 (PDF 18.9 KB)

Supplementary Material 4 (PDF 107 KB)

Supplementary Material 5 (PDF 107 KB)
