# Supplementary material for: Changes in the Fraction Metabolized in Children Younger than Four Years; When is Clearance Scaling for the Dominant Elimination Route in Adults Appropriate?
Source: Pharm Res. 2025 Oct 14;42(10):1691–700. doi: 10.1007/s11095-025-03948-7 (PMC12592267; doi:10.1007/s11095-025-03948-7)
Supplement: Supplementary file 1 — Supplementary Material 1 (DOCX 99.8 KB) [file 11095_2025_3948_MOESM1_ESM.docx]

**Changes in the fraction metabolized in children younger than four years; when is clearance scaling for the dominant elimination route in adults appropriate?**

Anne van Rongen^1^; Robbin Grijseels^1^; Elisa A.M. Calvier^2^; Karel Allegaert^3,4,5^; Catherijne A.J. Knibbe^1,6^; Elke H.J. Krekels^1,7^

1. Division of Systems Pharmacology and Pharmacy, Leiden Academic Center for Drug Research, Leiden University, Leiden, the Netherlands

2. Pharmacokinetics-Dynamics and Metabolism, Translational Medicine and Early Development, Sanofi R&D, Montpellier, France.

3. Department of Development and Regeneration, KU Leuven, Leuven, Belgium.

4. Department of Pharmaceutical and Pharmacological Sciences, KU Leuven, Leuven, Belgium.

5. Department of Hospital Pharmacy, Erasmus MC, Rotterdam, Netherlands.

6. Department of Clinical Pharmacy, St. Antonius Hospital, Nieuwegein and Utrecht, The Netherlands.

7. Certara Inc, Princeton, NJ, USA

Corresponding author: Elke H.J. Krekels ([elke.krekels@certara.com](mailto:elke.krekels@certara.com))

**Supplemental table 1.** Overview of model parameters used for the typical individuals in the virtual population.

| Parameter value [unit] | Typical individuals | | | | | | | |
| --- | --- | --- | --- | --- | --- | --- | --- | --- |
| Age | 1 day | 2 weeks | 1 month | 6 months | 1 year | 2 years | 4 years | 25 years |
| PMA [weeks] | 40.1 | 42.1 | 44.3 | 66.0 | 92.0 | 144.0 | 248.0 | 1340.0 |
| Bodyweight [kg] ^a^ | 3.47 | 3.90 | 4.29 | 7.57 | 9.92 | 12.37 | 16.01 | 72.65 |
| Height  [cm] ^a^ | 49.6 | 52.2 | 54.1 | 66.1 | 74.7 | 85.7 | 101.5 | 172.3 |
| BSA [m^2^] ^b^ | 0.223 | 0.242 | 0.258 | 0.380 | 0.461 | 0.548 | 0.665 | 1.857 |
| HSA [g/L] ^c^ | 33.7 | 36.8 | 37.6 | 39.6 | 40.4 | 41.2 | 42.0 | 44.0 |
| AAG [g/L] ^c^ | 0.269 | 0.487 | 0.545 | 0.6730 | 0.713 | 0.747 | 0.775 | 0.828 |
| Liver weight [g] ^c^ | 133.2 | 147.0 | 158.6 | 249.6 | 313.3 | 384.2 | 482.7 | 1614.2 |
| MPPGL ^d^ | 25.53 | 25.57 | 25.60 | 25.99 | 26.45 | 27.36 | 29.12 | 39.79 |
| hematocrit [%] ^d^ | 51.9 | 41.7 | 38.1 | 35.1 | 35.8 | 36.8 | 36.8 | 40.7 |
| Qh  [mL/min] ^c^ | 142.8 | 155.7 | 166.6 | 252.3 | 316.6 | 401.7 | 548.7 | 1705.0 |
| GFR [mL/min] ^e^ | 4.30 | 5.21 | 6.20 | 17.6 | 27.4 | 35.9 | 43.8 | 114.7 |
| MF [%] ^f^ | | | | | | | | |
| CYP1A2 ^d^ | 24 | 28 | 35 | 118 | 150 | 164 | 166 | 100 |
| CYP2A6 ^d^ | 2.1∙10^-9^ | 1.1∙10^-2^ | 0.48 | 99 | 100 | 100 | 100 | 100 |
| CYP2B6 ^d^ | 15 | 17 | 19 | 34 | 47 | 62 | 78 | 100 |
| CYP2C8 ^d^ | 38 | 77 | 86 | 97 | 99 | 99 | 100 | 100 |
| CYP2C9 ^d^ | 40 | 68 | 74 | 87 | 90 | 92 | 100 | 100 |
| CYP2C18 ^d^ | 30 | 31 | 33 | 84 | 95 | 97 | 98 | 100 |
| CYP2C19 ^d^ | 30 | 31 | 33 | 84 | 95 | 97 | 98 | 100 |
| CYP2D6 ^d^ | 6 | 32 | 47 | 84 | 91 | 95 | 98 | 100 |
| CYP2E1 ^d^ | 10 | 29 | 37 | 59 | 67 | 74 | 80 | 100 |
| CYP3A4 ^d^ | 11 | 11 | 13 | 48 | 78 | 96 | 103 | 100 |
| UGT1A1 ^d^ | 0.2 | 7 | 23 | 98 | 104 | 100 | 100 | 100 |
| UGT1A4 ^d^ | 74 | 74 | 74 | 74 | 75 | 77 | 80 | 100 |
| UGT1A6 ^d^ | 15 | 23 | 30 | 63 | 76 | 87 | 94 | 100 |
| UGT1A9 ^d^ | 9 | 10 | 12 | 34 | 52 | 71 | 86 | 100 |
| UGT2B7 ^d^ | 8 | 9 | 9 | 11 | 13 | 18 | 27 | 100 |
| SULT1A1 ^g^ | 100 | 100 | 100 | 100 | 100 | 100 | 100 | 100 |
| AGP = concentration α1-acid glycoprotein; BSA = body surface area; HSA = concentration human serum albumin; MPPGL = milligram protein per gram of liver; PMA = post menstrual age; Qh = hepatic blood flow; | | | | | | | | |

^a^ Average value from males and females from CDC growth chart

^b^ According to Haycock *et al*. (J Pediatr. 1978 ;93(1) : 62-6) for children ≤15 kg and to Du Bois and Du Bois (Arch Intern Med. 1916; 17:863-871) for heavier individuals

^c^ According to Johnson *et al*. Clin Pharmacokinet. 2006; 45(9) : 931-56

^d^ According to Simcyp® V15.R1 library

^e^ According to Salem *et al*. Clin. Pharmacokinet. 2014; 53 : 625–36

^f^ MF is expressed as percentage of adult microsomal unbound intrinsic clearance

^g^ According to Hines (Pharmacol Ther. 2008 ;118(2) : 250-67)

**
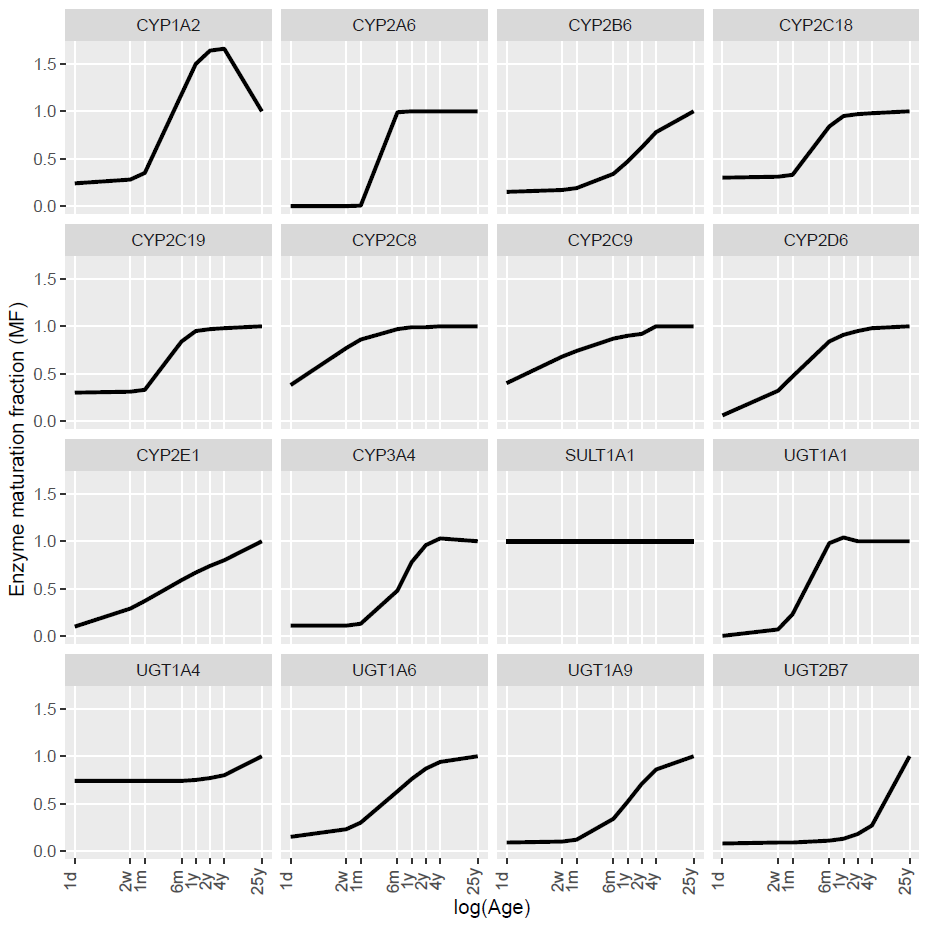
**

**Supplemental figure 1.** Maturation pattens of the iso-enzymes included in the analysis. The enzyme maturation functions (MF) are expressed as fraction of enzyme activity per gram of microsomal protein in adults. All maturation patterns are according to the Simcyp® V15.R1 library, except for SULT1A1 which is taken from Hines (Pharmacol Ther. 2008 ;118(2) : 250-67)
