# Supplementary material for: Changes in the Fraction Metabolized in Children Younger than Four Years; When is Clearance Scaling for the Dominant Elimination Route in Adults Appropriate?
Source: Pharm Res. 2025 Oct 14;42(10):1691–700. doi: 10.1007/s11095-025-03948-7 (PMC12592267; doi:10.1007/s11095-025-03948-7)
Supplement: Supplementary file 2 — Supplementary Material 2 (PDF 19.0 KB) [file 11095_2025_3948_MOESM2_ESM.pdf]

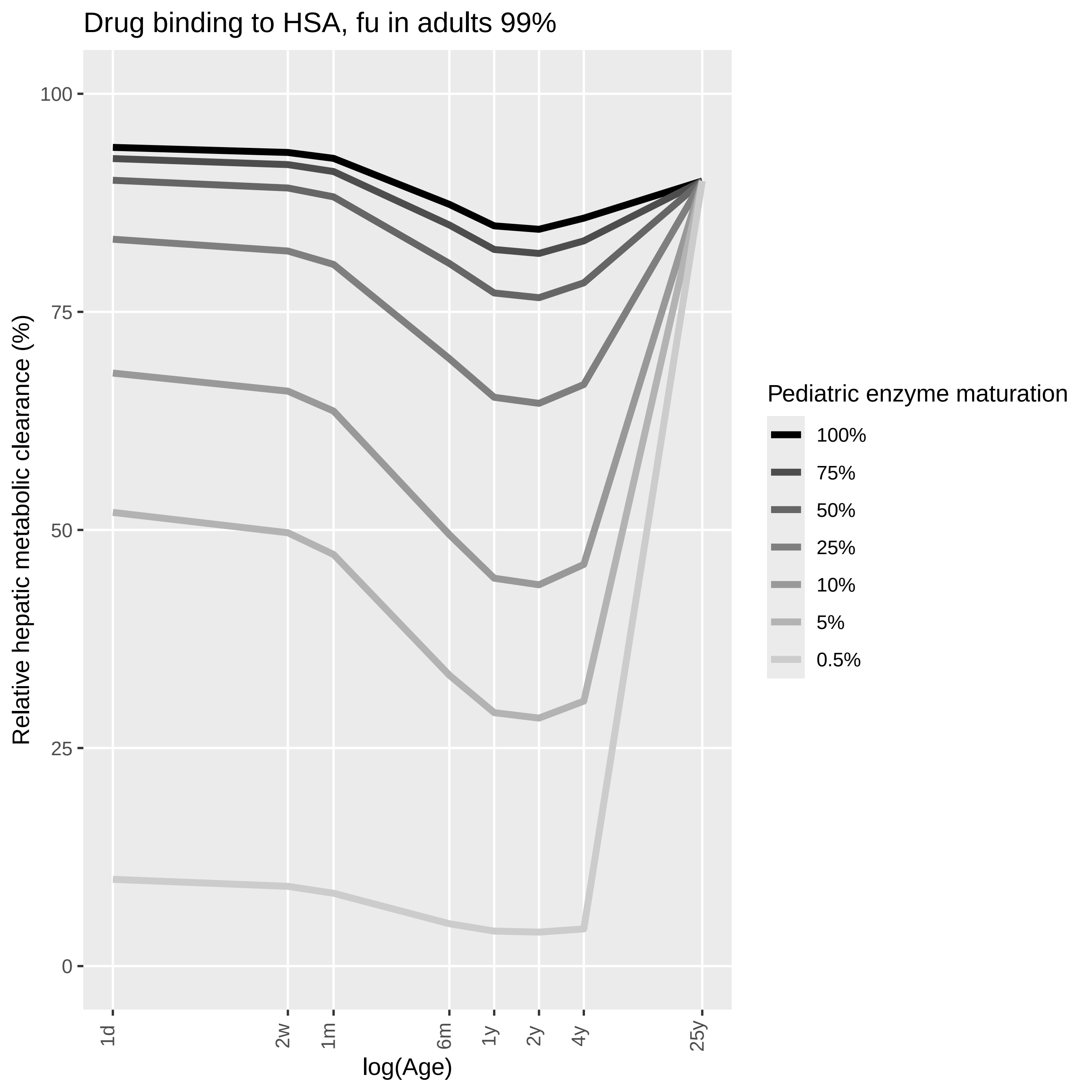

Drug binding to HSA, fu in adults 99%
Relative hepatic metabolic clearance (%)
100
75
50
25
0
1d
2w
1m
6m
1y
2y
4y
25y
log(Age)
Pediatric enzyme maturation
100%
75%
50%
25%
10%
5%
0.5%

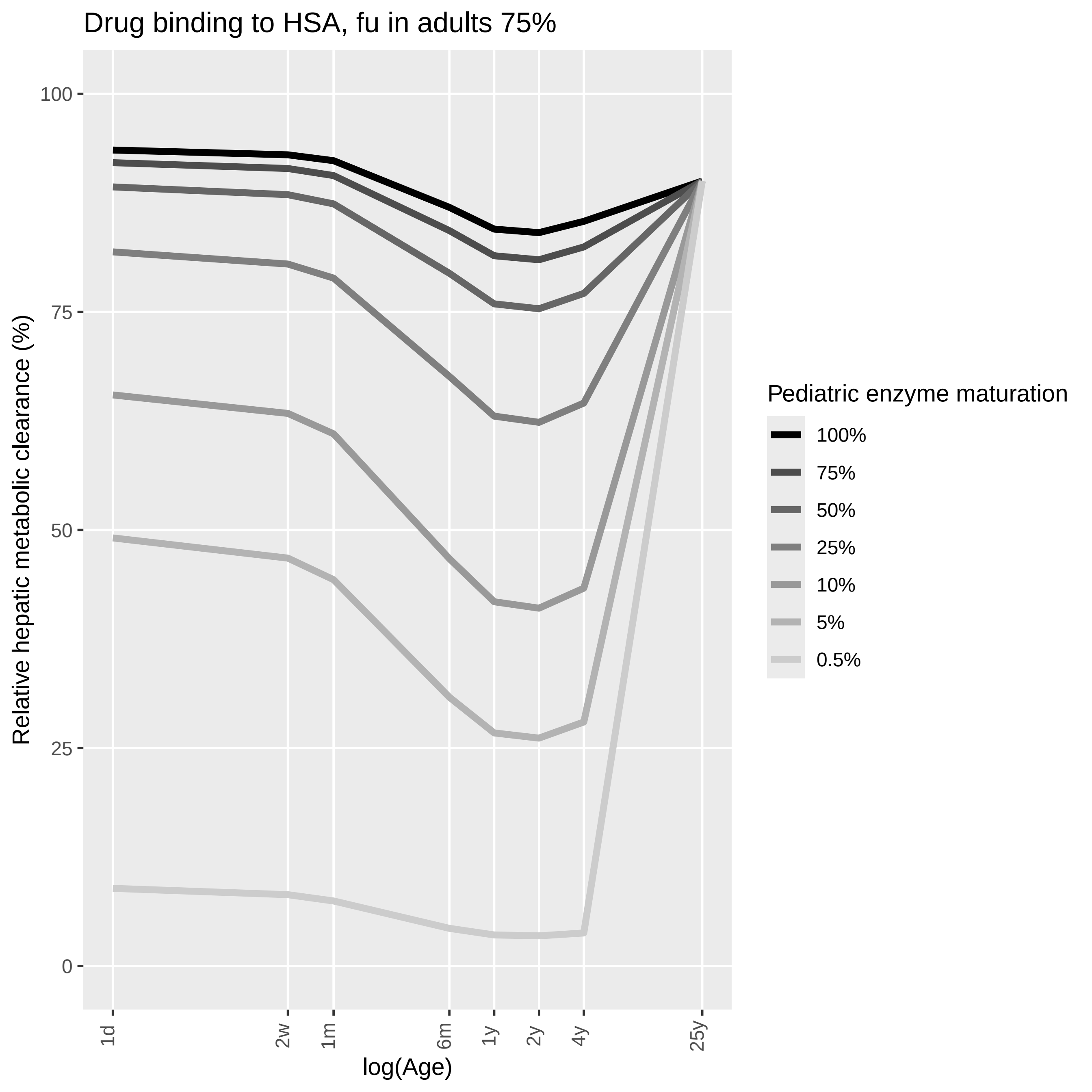

Drug binding to HSA, fu in adults 75%
Relative hepatic metabolic clearance (%)
100
75
50
25
0
1d
2w
1m
6m
1y
2y
4y
25y
log(Age)
Pediatric enzyme maturation
100%
75%
50%
25%
10%
5%
0.5%

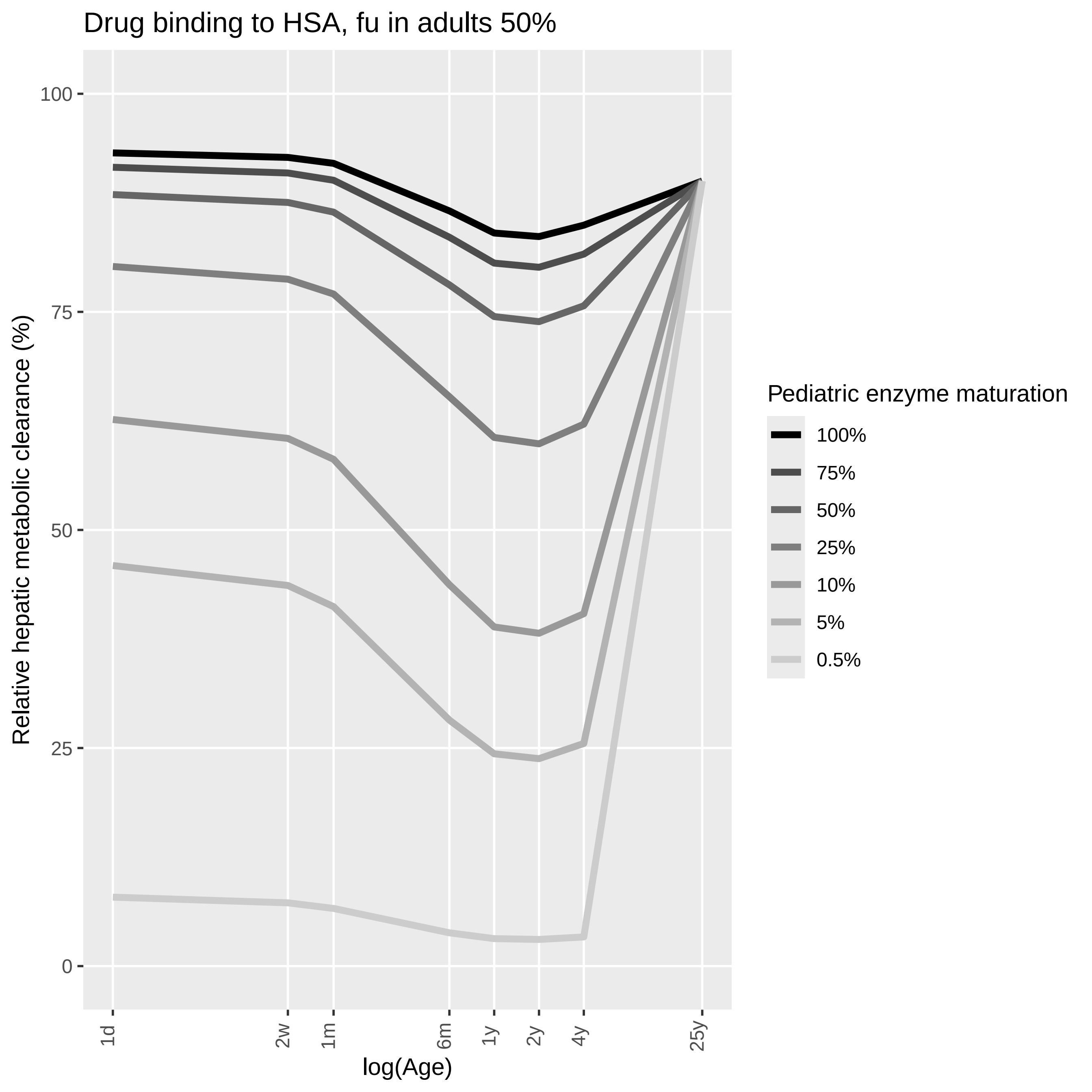
Drug binding to HSA, fu in adults 50%
Relative hepatic metabolic clearance (%)
100
75
50
25
0
1d
2w
1m
6m
1y
2y
4y
25y
log(Age)
Pediatric enzyme maturation
100%
75%
50%
25%
10%
5%
0.5%

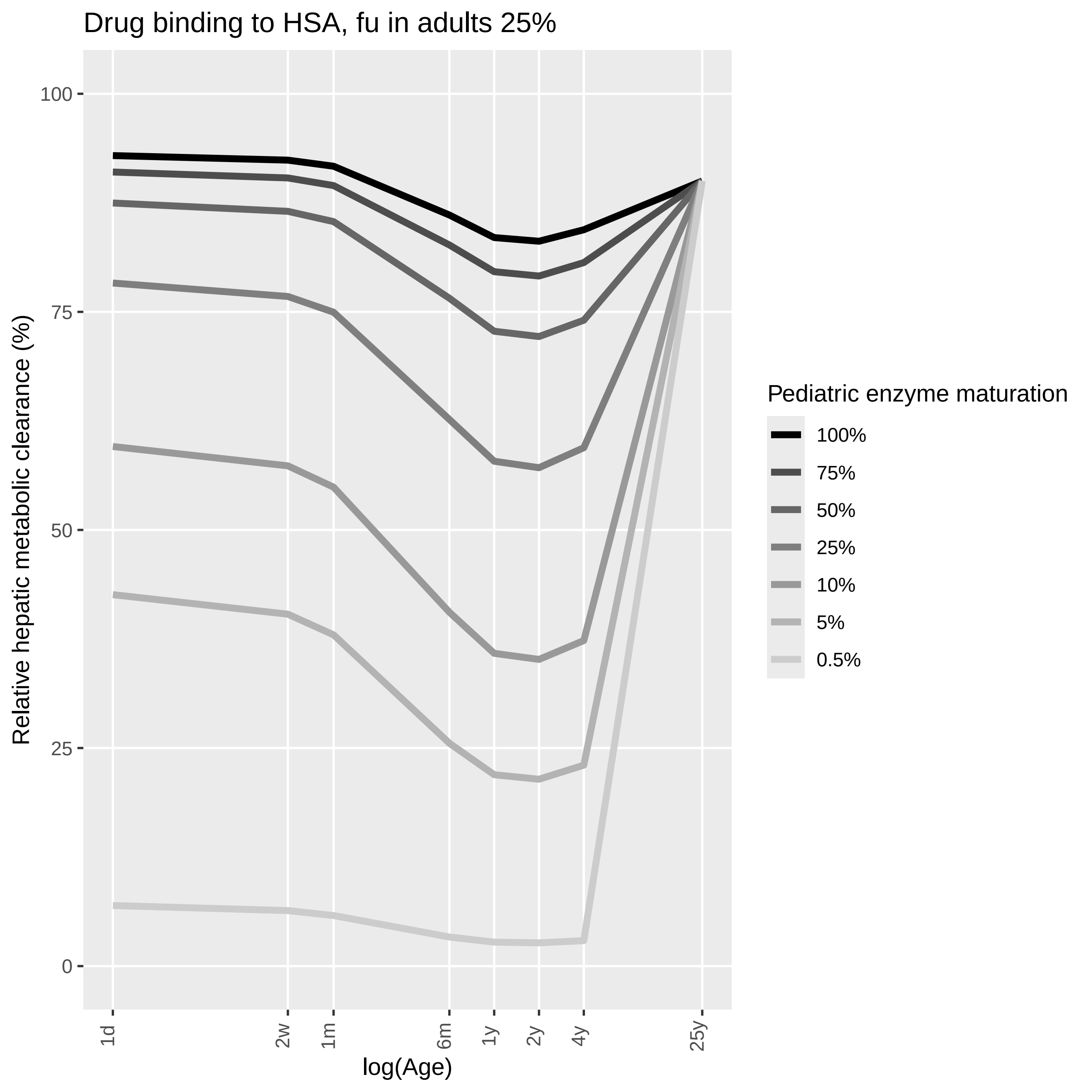
Drug binding to HSA, fu in adults 25%
Relative hepatic metabolic clearance (%)
100
75
50
25
0
1d
2w
1m
6m
1y
2y
4y
25y
log(Age)
Pediatric enzyme maturation
100%
75%
50%
25%
10%
5%
0.5%

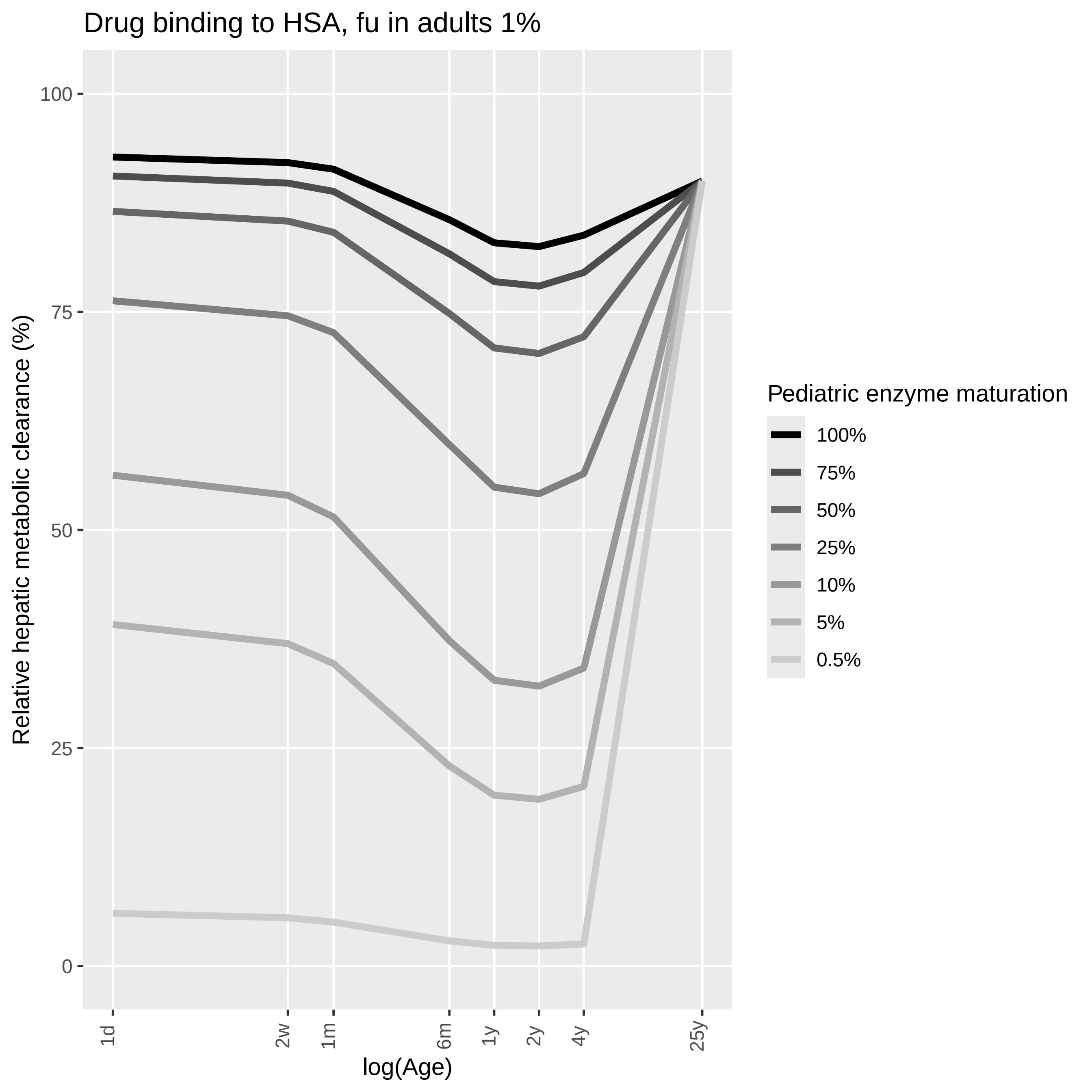
Drug binding to HSA, fu in adults 1%
Relative hepatic metabolic clearance (%)
100
75
50
25
0
1d
2w
1m
6m
1y
2y
4y
25y
log(Age)
Pediatric enzyme maturation
100%
75%
50%
25%
10%
5%
0.5%

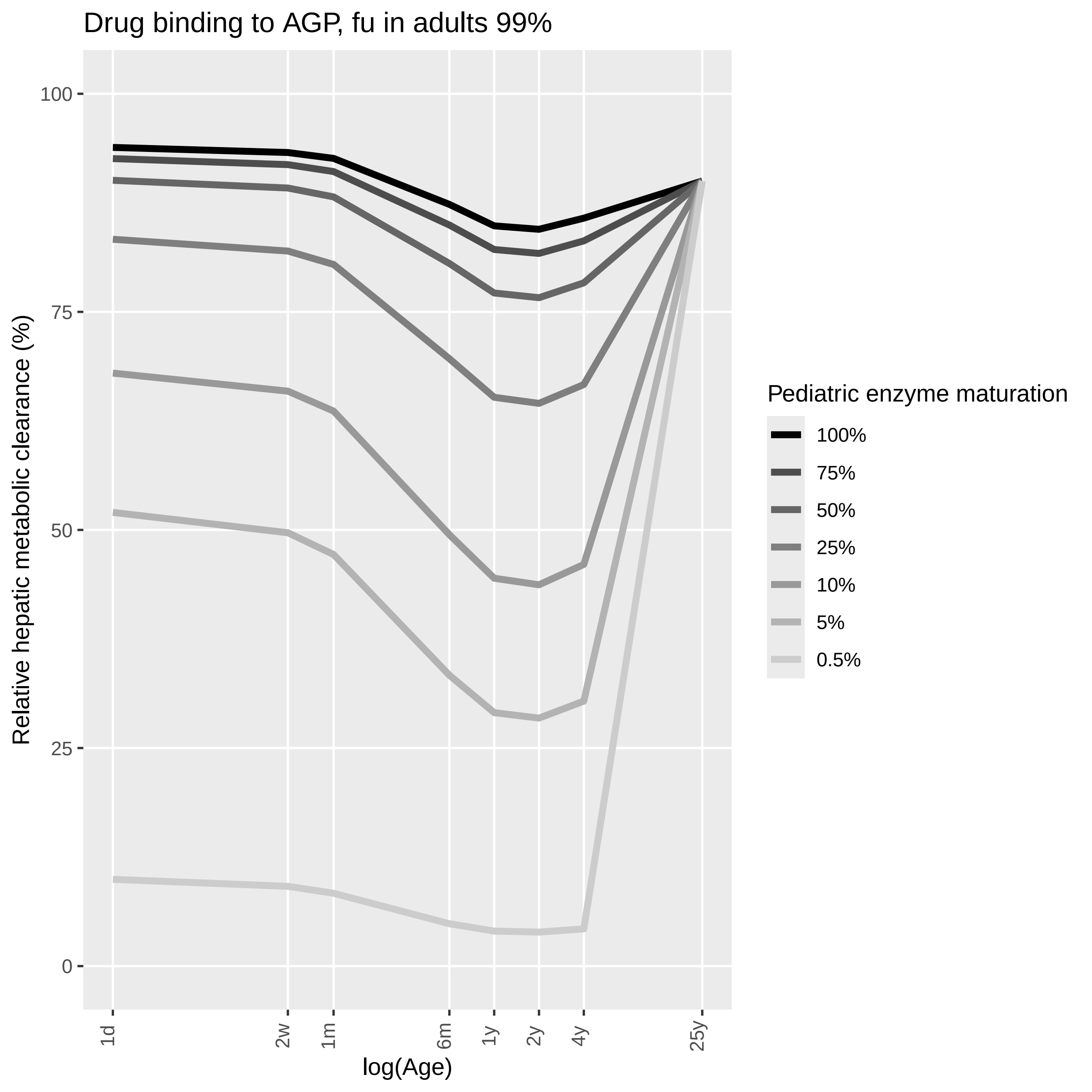

Drug binding to AGP, fu in adults 99%
Relative hepatic metabolic clearance (%)
100
75
50
25
0
1d
2w
1m
6m
1y
2y
4y
25y
log(Age)
Pediatric enzyme maturation
100%
75%
50%
25%
10%
5%
0.5%

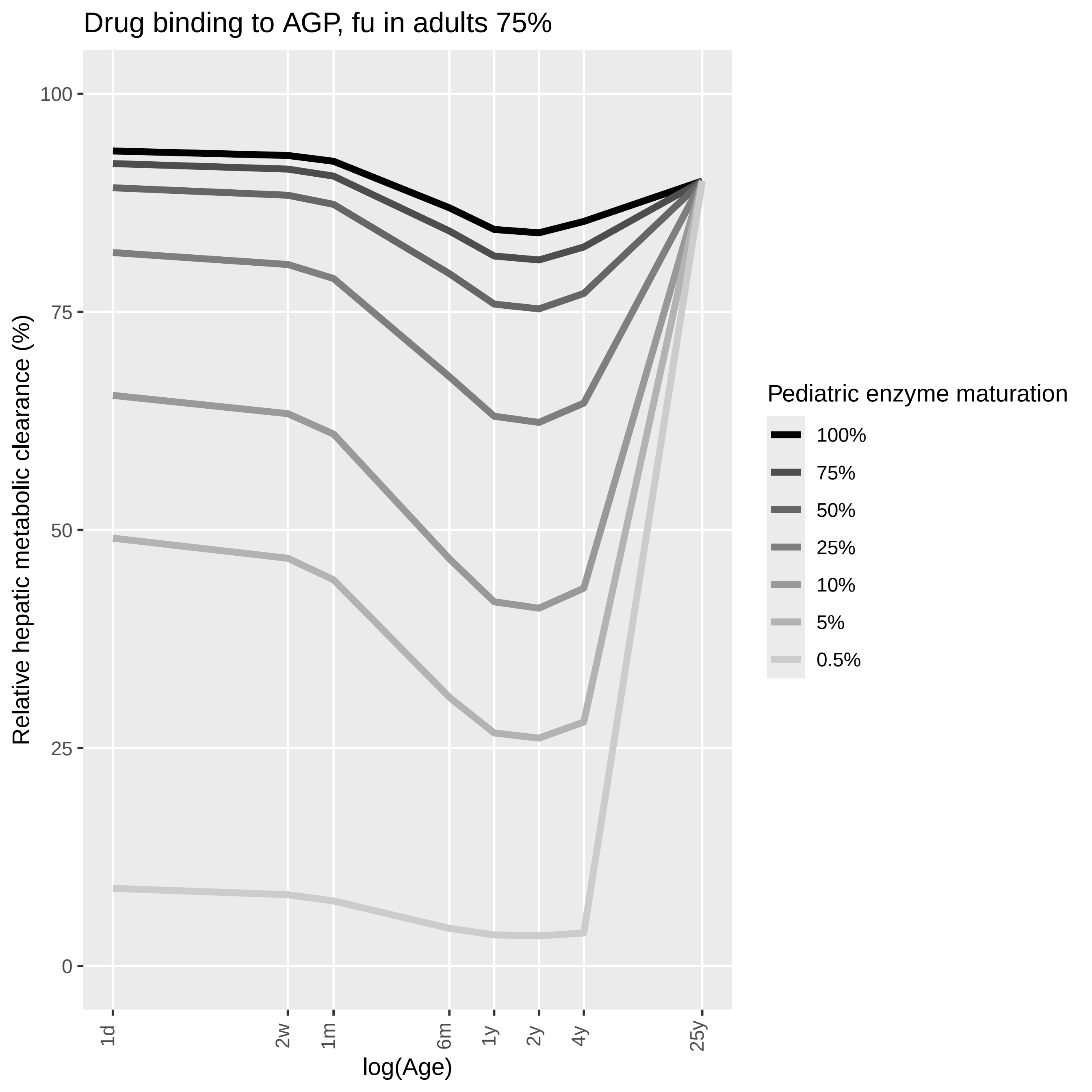
Drug binding to AGP, fu in adults 75%
Relative hepatic metabolic clearance (%)
100
75
50
25
0
1d
2w
1m
6m
1y
2y
4y
25y
log(Age)
Pediatric enzyme maturation
100%
75%
50%
25%
10%
5%
0.5%

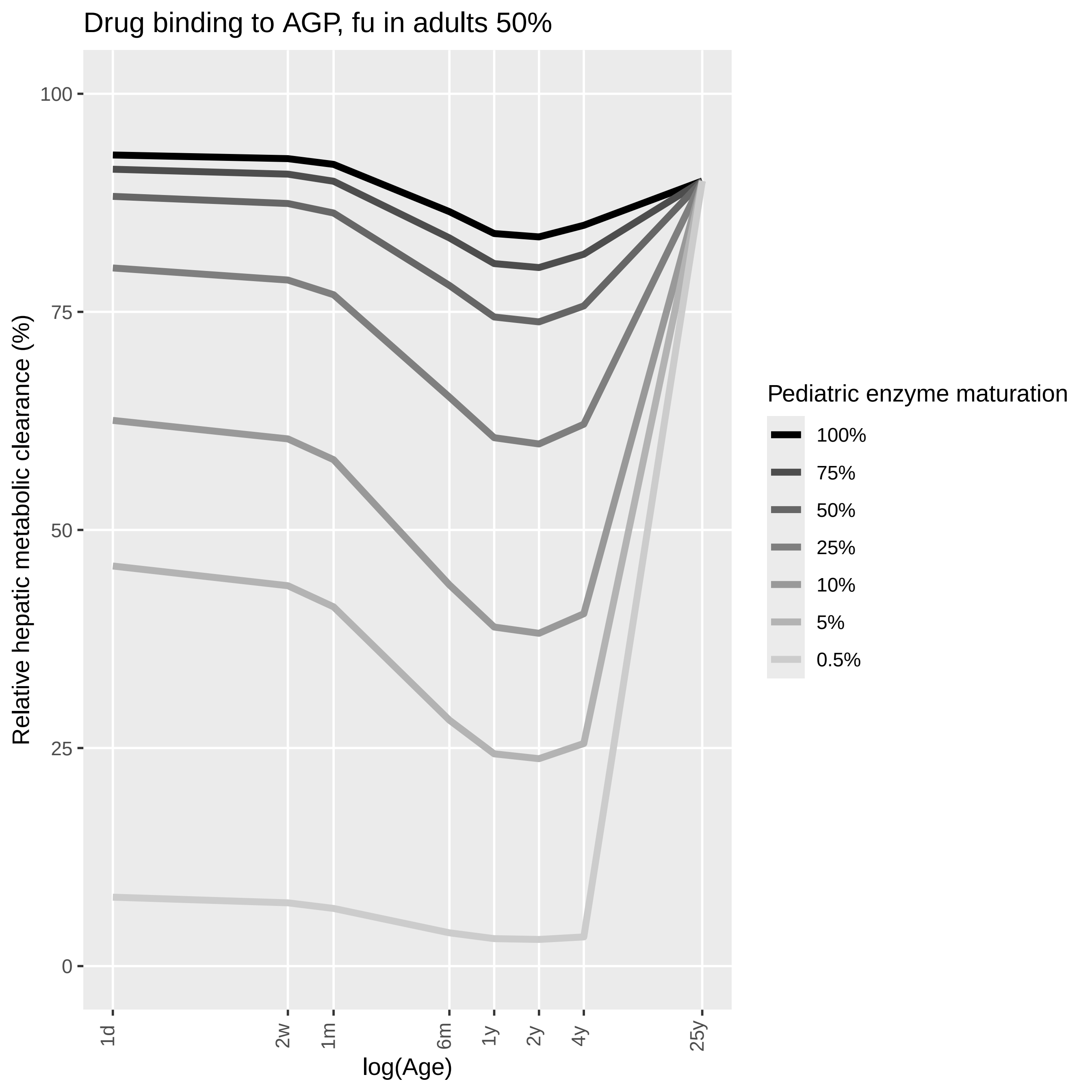

Drug binding to AGP, fu in adults 50%
Relative hepatic metabolic clearance (%)
100
75
50
25
0
1d
2w
1m
6m
1y
2y
4y
25y
log(Age)
Pediatric enzyme maturation
100%
75%
50%
25%
10%
5%
0.5%

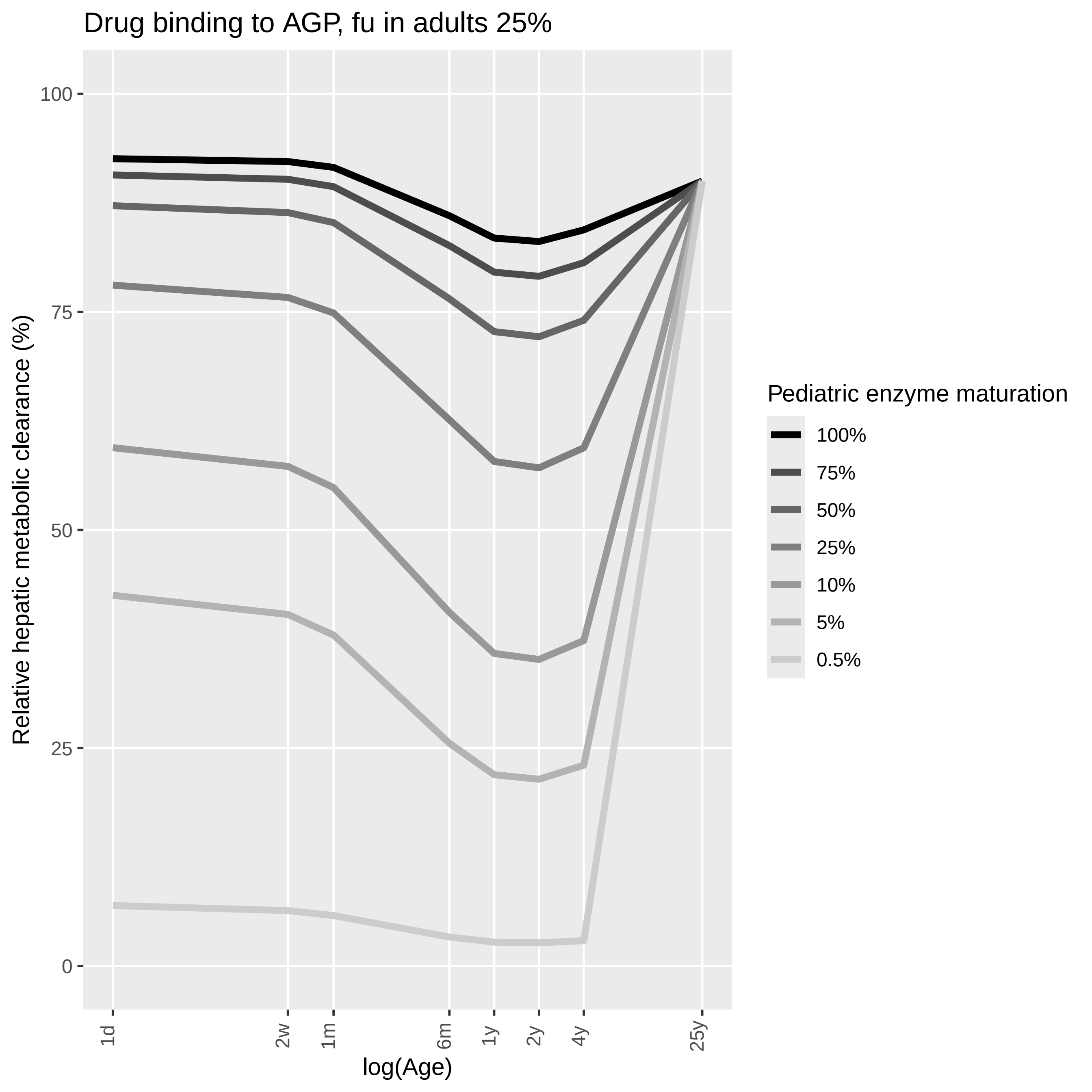
Drug binding to AGP, fu in adults 25%
Relative hepatic metabolic clearance (%)
100
75
50
25
0
1d
2w
1m
6m
1y
2y
4y
25y
log(Age)
Pediatric enzyme maturation
100%
75%
50%
25%
10%
5%
0.5%

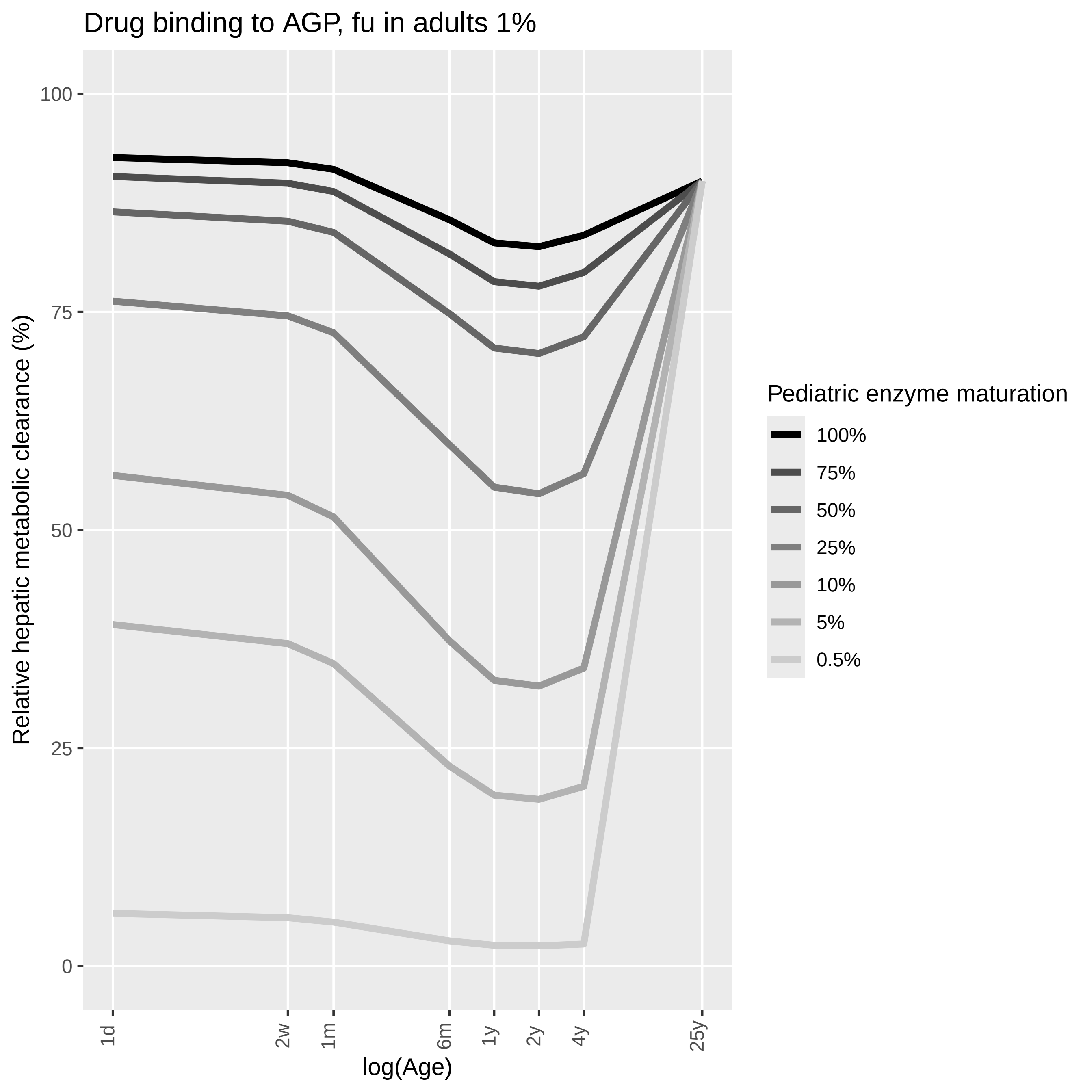

Drug binding to AGP, fu in adults 1%
Relative hepatic metabolic clearance (%)
100
75
50
25
0
1d
2w
1m
6m
1y
2y
4y
25y
log(Age)
Pediatric enzyme maturation
100%
75%
50%
25%
10%
5%
0.5%
