# Supplementary material for: Changes in the Fraction Metabolized in Children Younger than Four Years; When is Clearance Scaling for the Dominant Elimination Route in Adults Appropriate?
Source: Pharm Res. 2025 Oct 14;42(10):1691–700. doi: 10.1007/s11095-025-03948-7 (PMC12592267; doi:10.1007/s11095-025-03948-7)
Supplement: Supplementary file 5 — Supplementary Material 5 (PDF 107 KB) [file 11095_2025_3948_MOESM5_ESM.pdf]

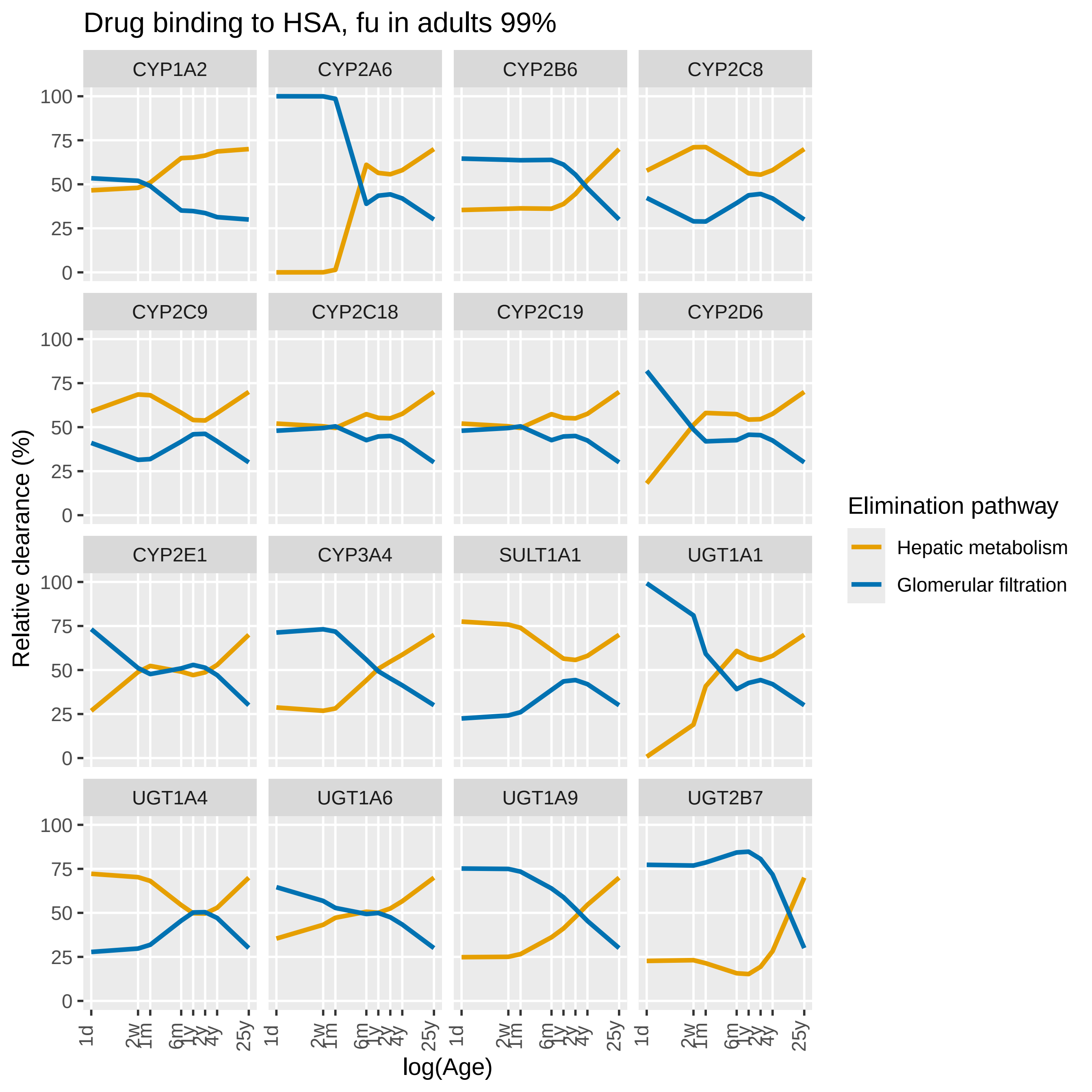

Drug binding to HSA, fu in adults 99%
CYP1A2
CYP2A6
CYP2B6
CYP2C8
CYP2C9
CYP2C18
CYP2C19
CYP2D6
CYP2E1
CYP3A4
SULT1A1
UGT1A1
UGT1A4
UGT1A6
UGT1A9
UGT2B7
Relative clearance (%)
0
25
50
75
100
1d
2w
1m
6m
1y
2y
4y
25y
log(Age)
Elimination pathway
Hepatic metabolism
Glomerular filtration

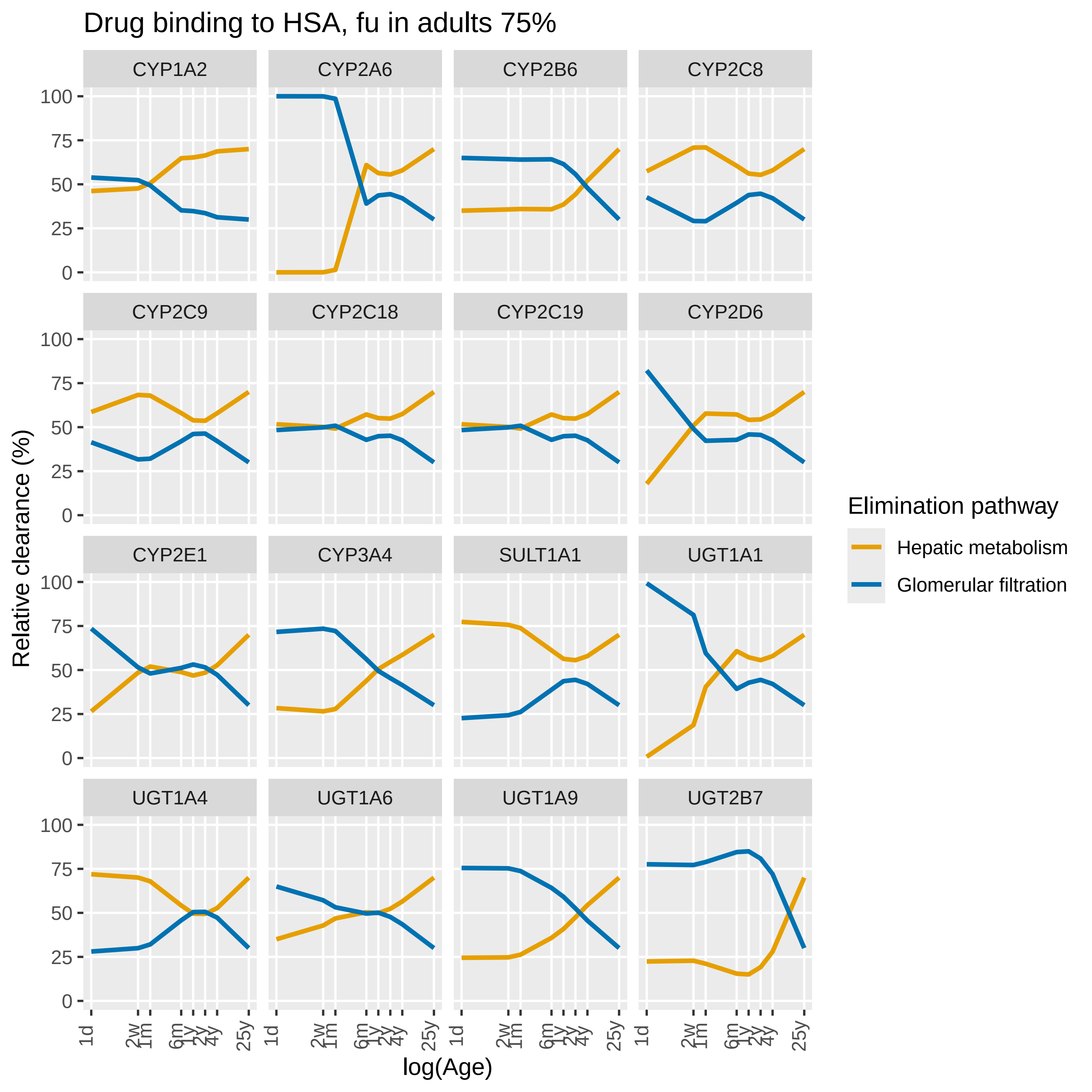
Drug binding to HSA, fu in adults 75%
CYP1A2
CYP2A6
CYP2B6
CYP2C8
CYP2C9
CYP2C18
CYP2C19
CYP2D6
CYP2E1
CYP3A4
SULT1A1
UGT1A1
UGT1A4
UGT1A6
UGT1A9
UGT2B7
Relative clearance (%)
0
25
50
75
100
1d
2w
1m
6m
1y
2y
4y
25y
log(Age)
Elimination pathway
Hepatic metabolism
Glomerular filtration

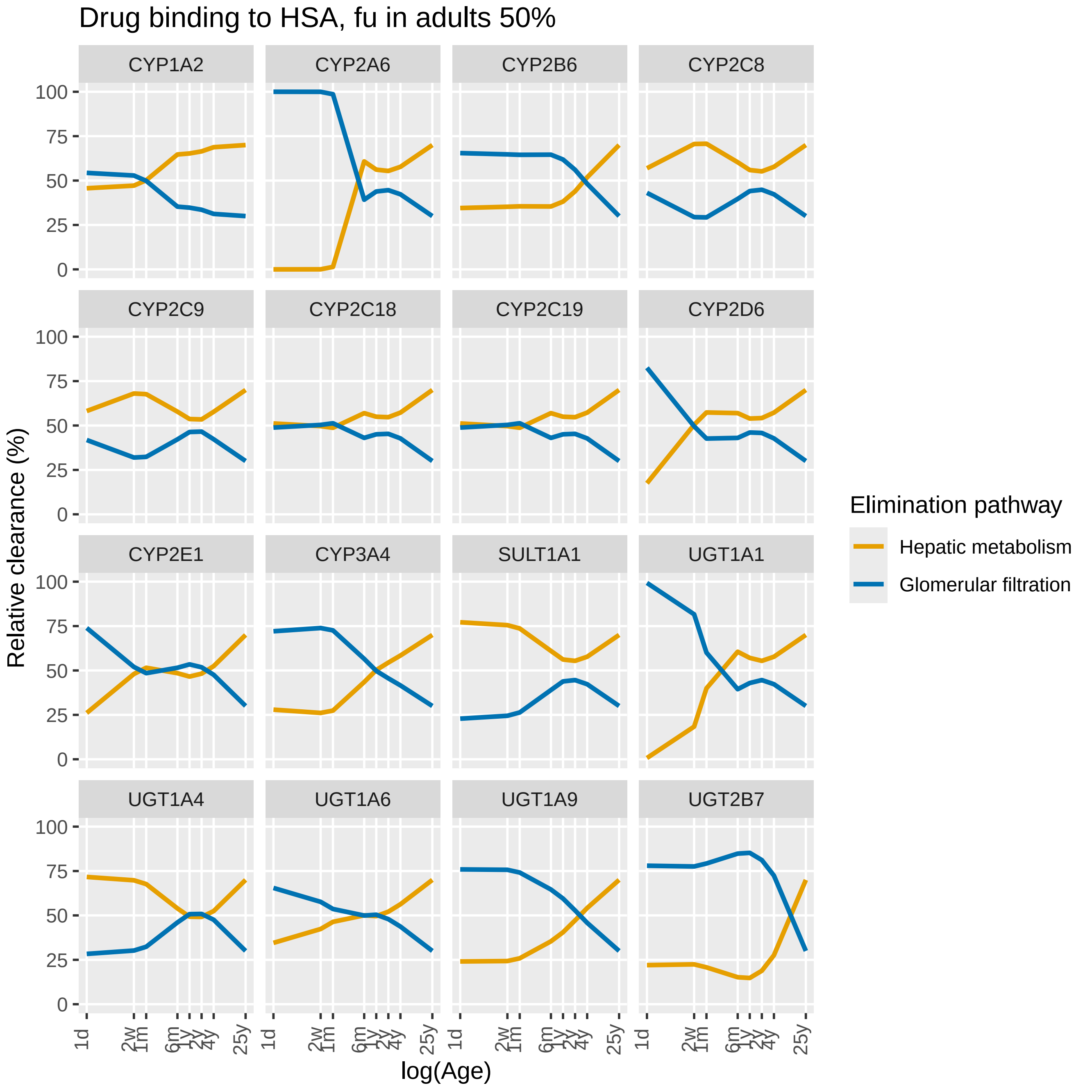

Drug binding to HSA, fu in adults 50%
CYP1A2
CYP2A6
CYP2B6
CYP2C8
CYP2C9
CYP2C18
CYP2C19
CYP2D6
CYP2E1
CYP3A4
SULT1A1
UGT1A1
UGT1A4
UGT1A6
UGT1A9
UGT2B7
Relative clearance (%)
0
25
50
75
100
1d
2w
1m
6m
1y
2y
4y
25y
log(Age)
Elimination pathway
Hepatic metabolism
Glomerular filtration

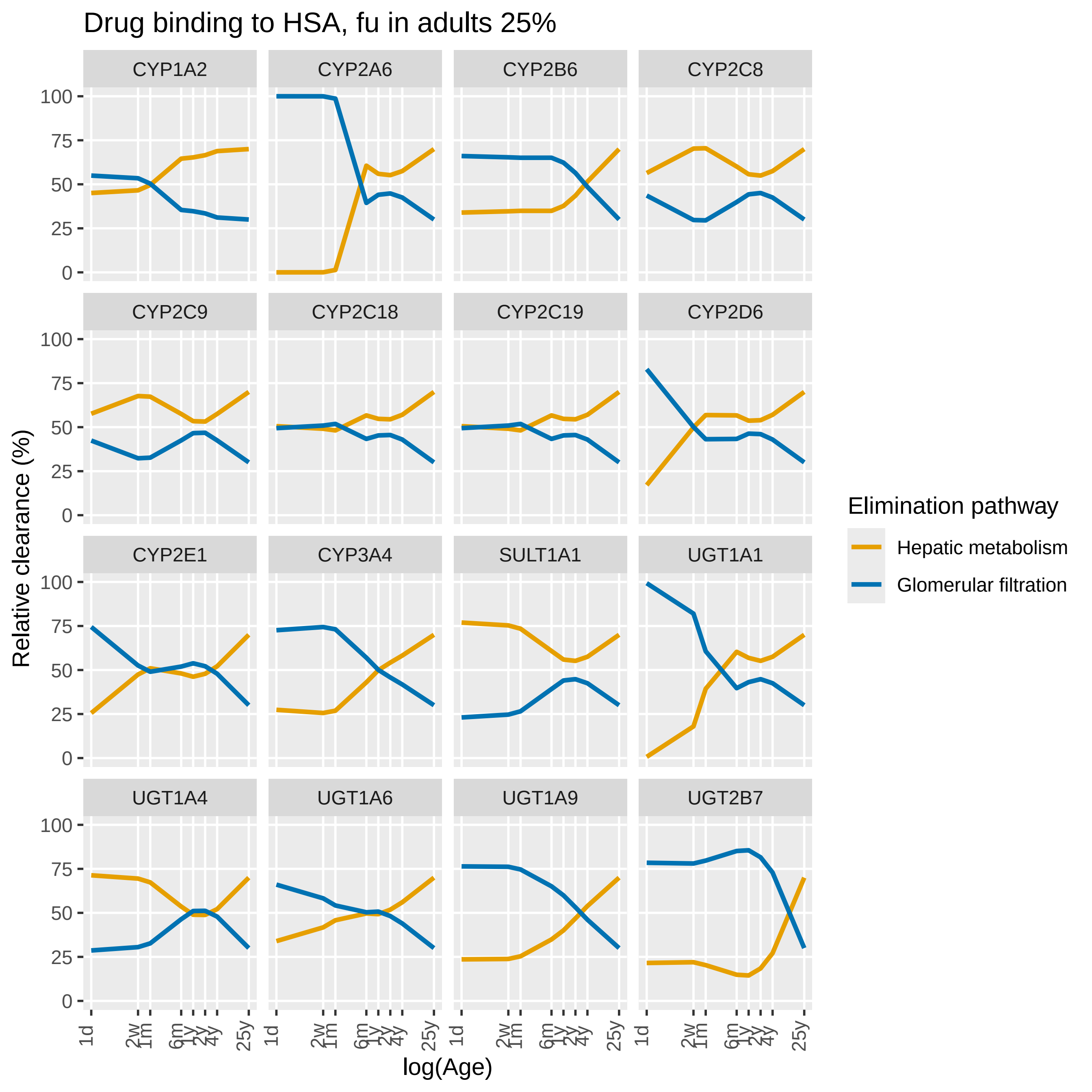
Drug binding to HSA, fu in adults 25%
CYP1A2
CYP2A6
CYP2B6
CYP2C8
CYP2C9
CYP2C18
CYP2C19
CYP2D6
CYP2E1
CYP3A4
SULT1A1
UGT1A1
UGT1A4
UGT1A6
UGT1A9
UGT2B7
Relative clearance (%)
0
25
50
75
100
log(Age)
1d
2w
1m
6m
1y
2y
4y
25y
Elimination pathway
Hepatic metabolism
Glomerular filtration

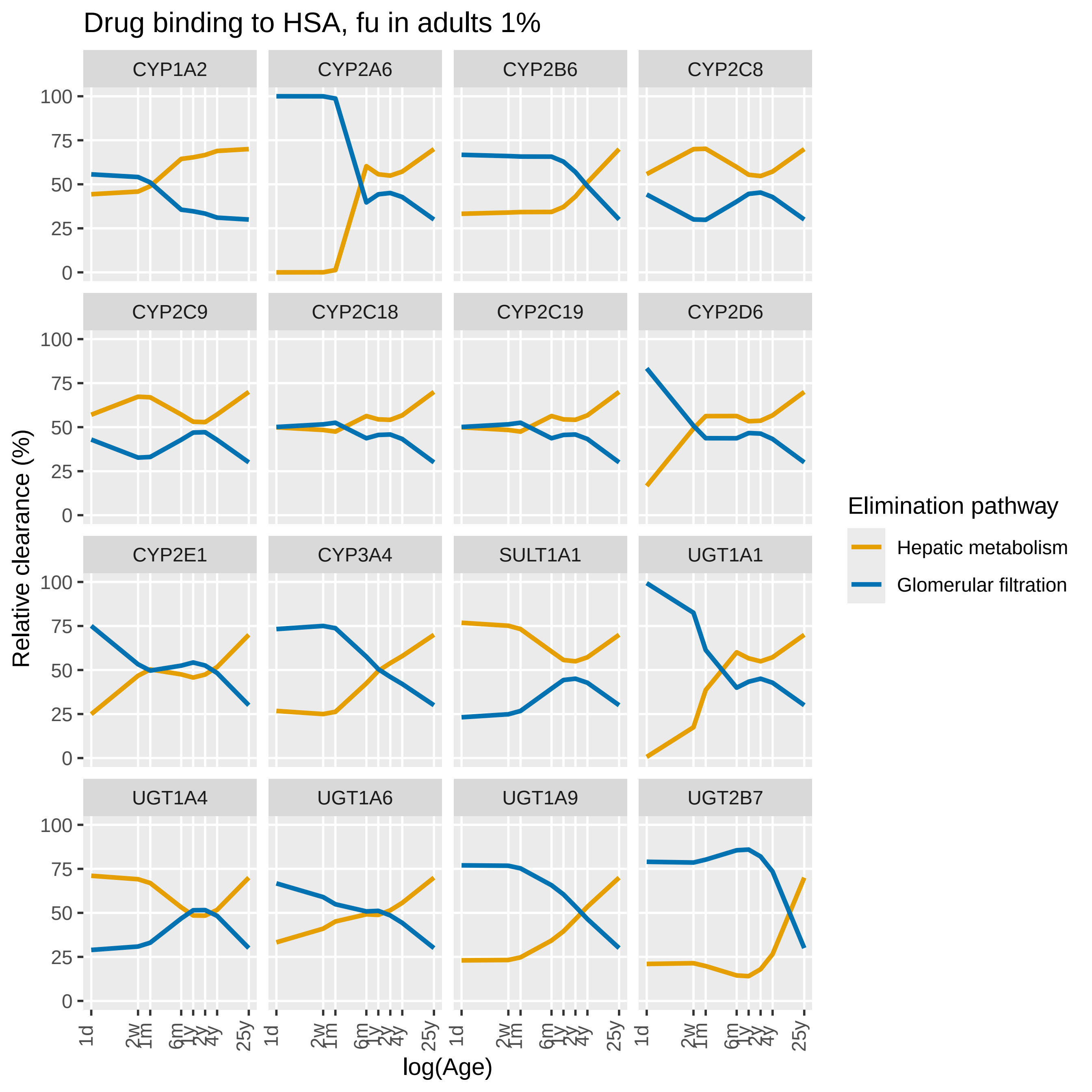

Drug binding to HSA, fu in adults 1%
CYP1A2
CYP2A6
CYP2B6
CYP2C8
CYP2C9
CYP2C18
CYP2C19
CYP2D6
CYP2E1
CYP3A4
SULT1A1
UGT1A1
UGT1A4
UGT1A6
UGT1A9
UGT2B7
Relative clearance (%)
0
25
50
75
100
1d
2w
1m
6m
1y
2y
4y
25y
log(Age)
Elimination pathway
Hepatic metabolism
Glomerular filtration

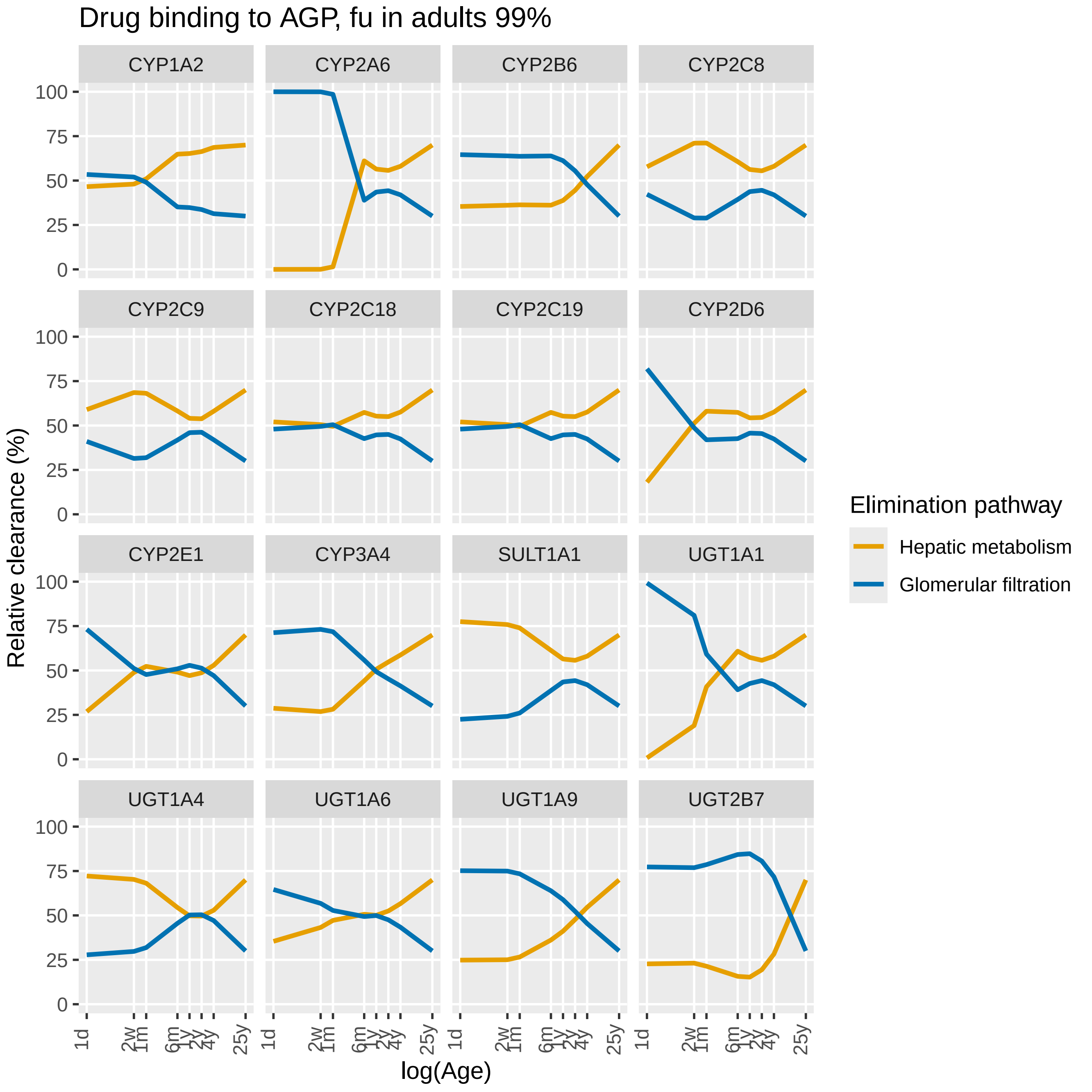
Drug binding to AGP, fu in adults 99%
CYP1A2
CYP2A6
CYP2B6
CYP2C8
CYP2C9
CYP2C18
CYP2C19
CYP2D6
CYP2E1
CYP3A4
SULT1A1
UGT1A1
UGT1A4
UGT1A6
UGT1A9
UGT2B7
Relative clearance (%)
0
25
50
75
100
log(Age)
1d
2w
1m
6m
1y
2y
4y
25y
Elimination pathway
Hepatic metabolism
Glomerular filtration

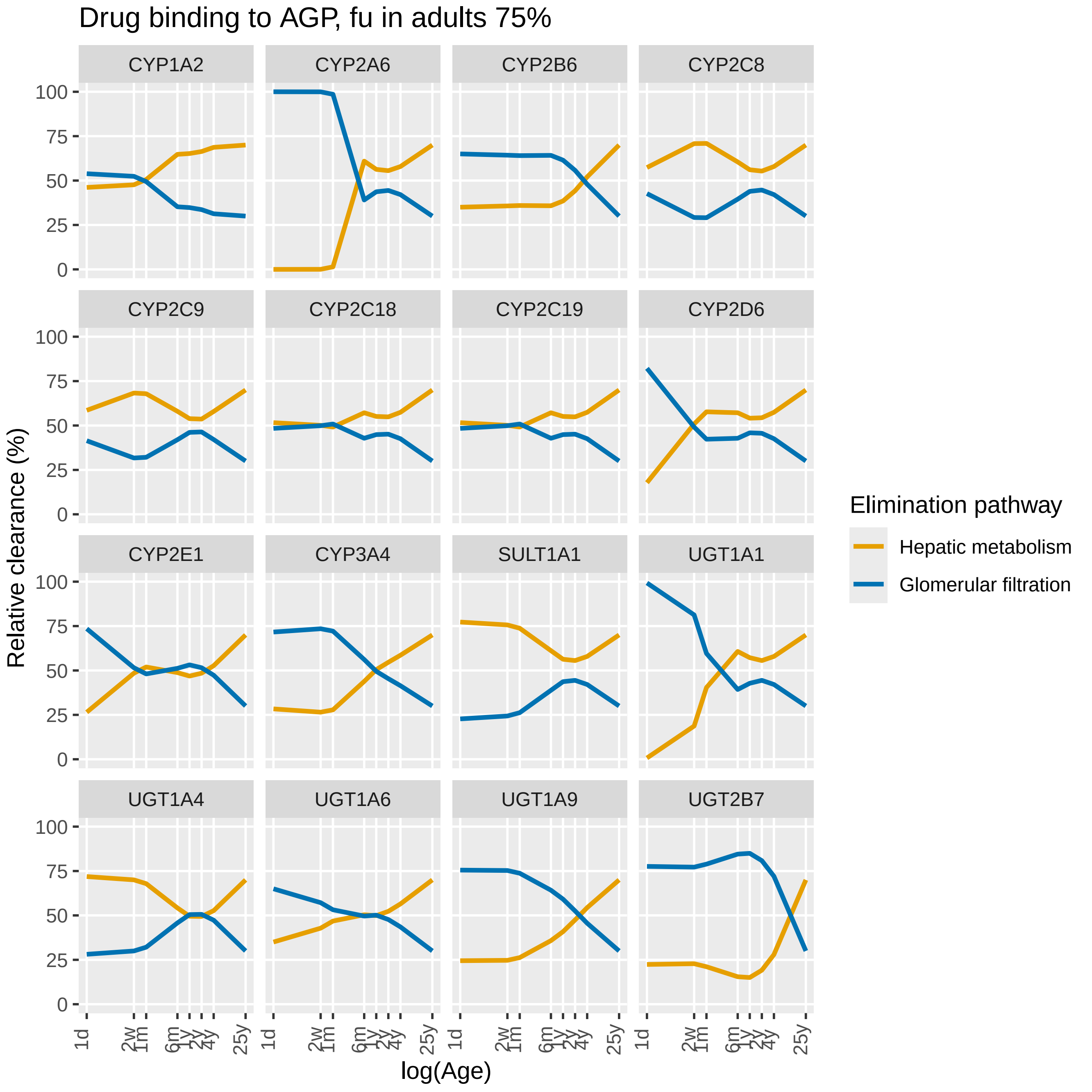

Drug binding to AGP, fu in adults 75%
CYP1A2
CYP2A6
CYP2B6
CYP2C8
CYP2C9
CYP2C18
CYP2C19
CYP2D6
CYP2E1
CYP3A4
SULT1A1
UGT1A1
UGT1A4
UGT1A6
UGT1A9
UGT2B7
Relative clearance (%)
0
25
50
75
100
log(Age)
1d
2w
1m
6m
1y
2y
4y
25y
Elimination pathway
Hepatic metabolism
Glomerular filtration

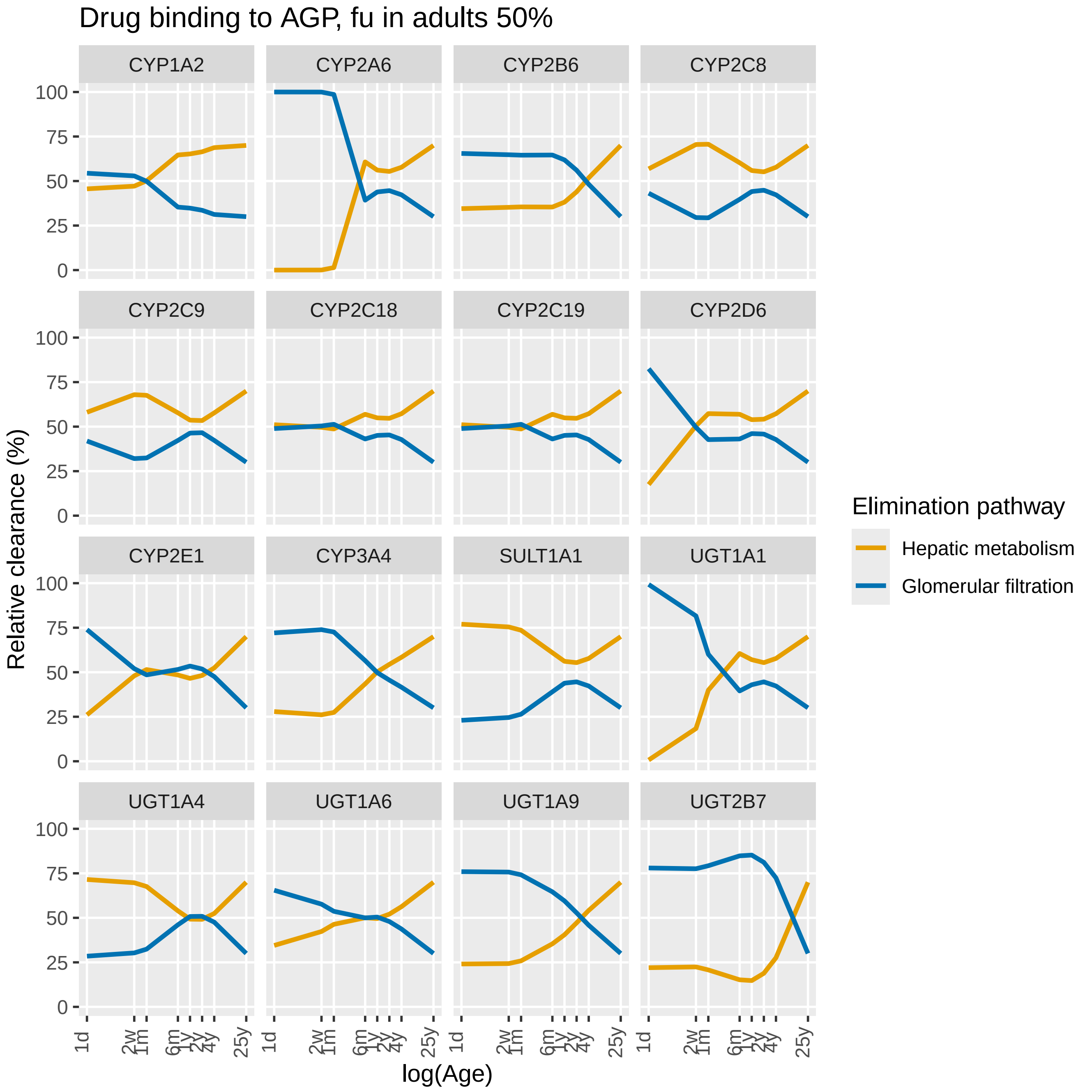
Drug binding to AGP, fu in adults 50%
CYP1A2
CYP2A6
CYP2B6
CYP2C8
CYP2C9
CYP2C18
CYP2C19
CYP2D6
CYP2E1
CYP3A4
SULT1A1
UGT1A1
UGT1A4
UGT1A6
UGT1A9
UGT2B7
Relative clearance (%)
0
25
50
75
100
log(Age)
1d
2w
1m
6m
1y
2y
4y
25y
Elimination pathway
Hepatic metabolism
Glomerular filtration

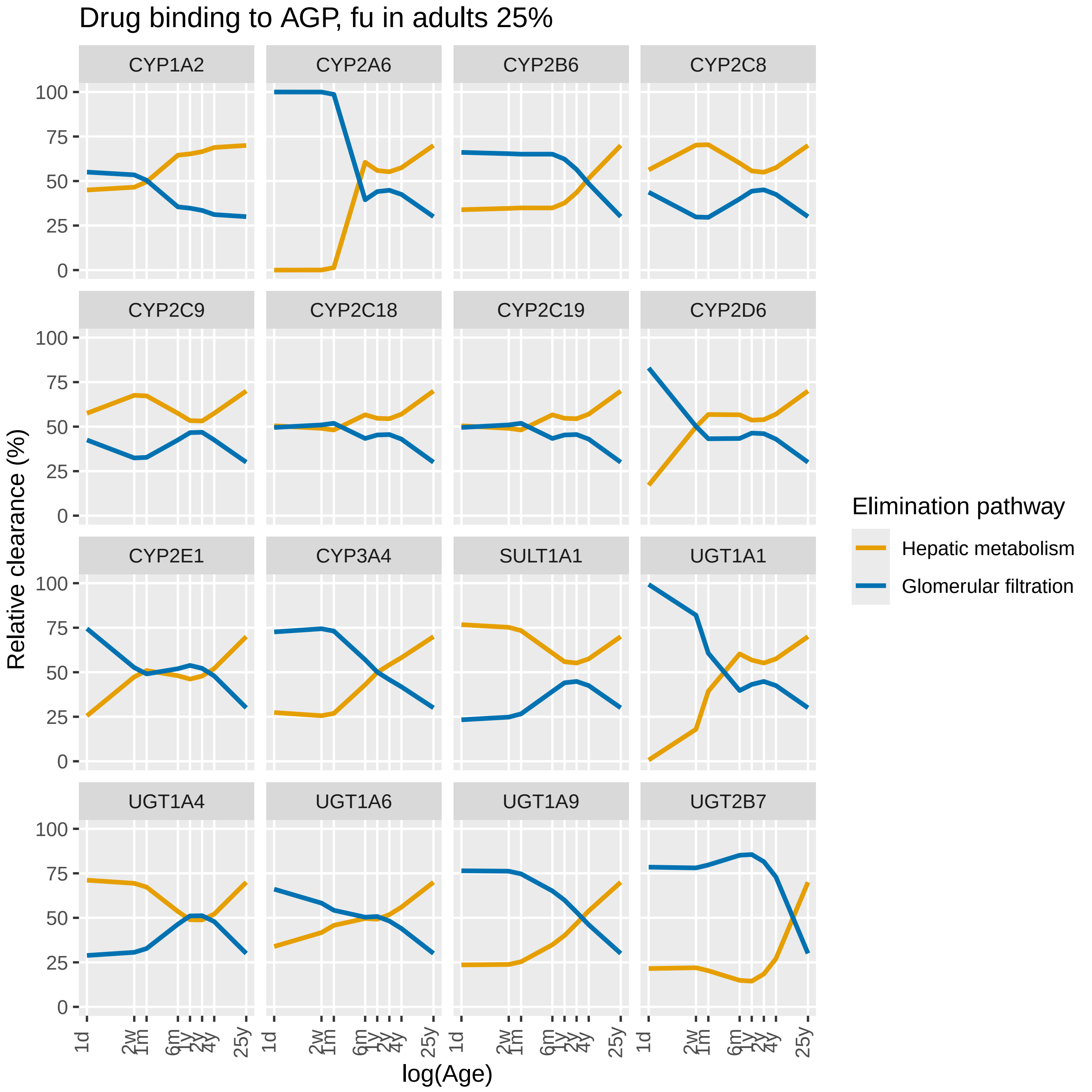
Drug binding to AGP, fu in adults 25%
CYP1A2
CYP2A6
CYP2B6
CYP2C8
CYP2C9
CYP2C18
CYP2C19
CYP2D6
CYP2E1
CYP3A4
SULT1A1
UGT1A1
UGT1A4
UGT1A6
UGT1A9
UGT2B7
Relative clearance (%)
0
25
50
75
100
1d
2w
1m
6m
1y
2y
4y
25y
log(Age)
Elimination pathway
Hepatic metabolism
Glomerular filtration

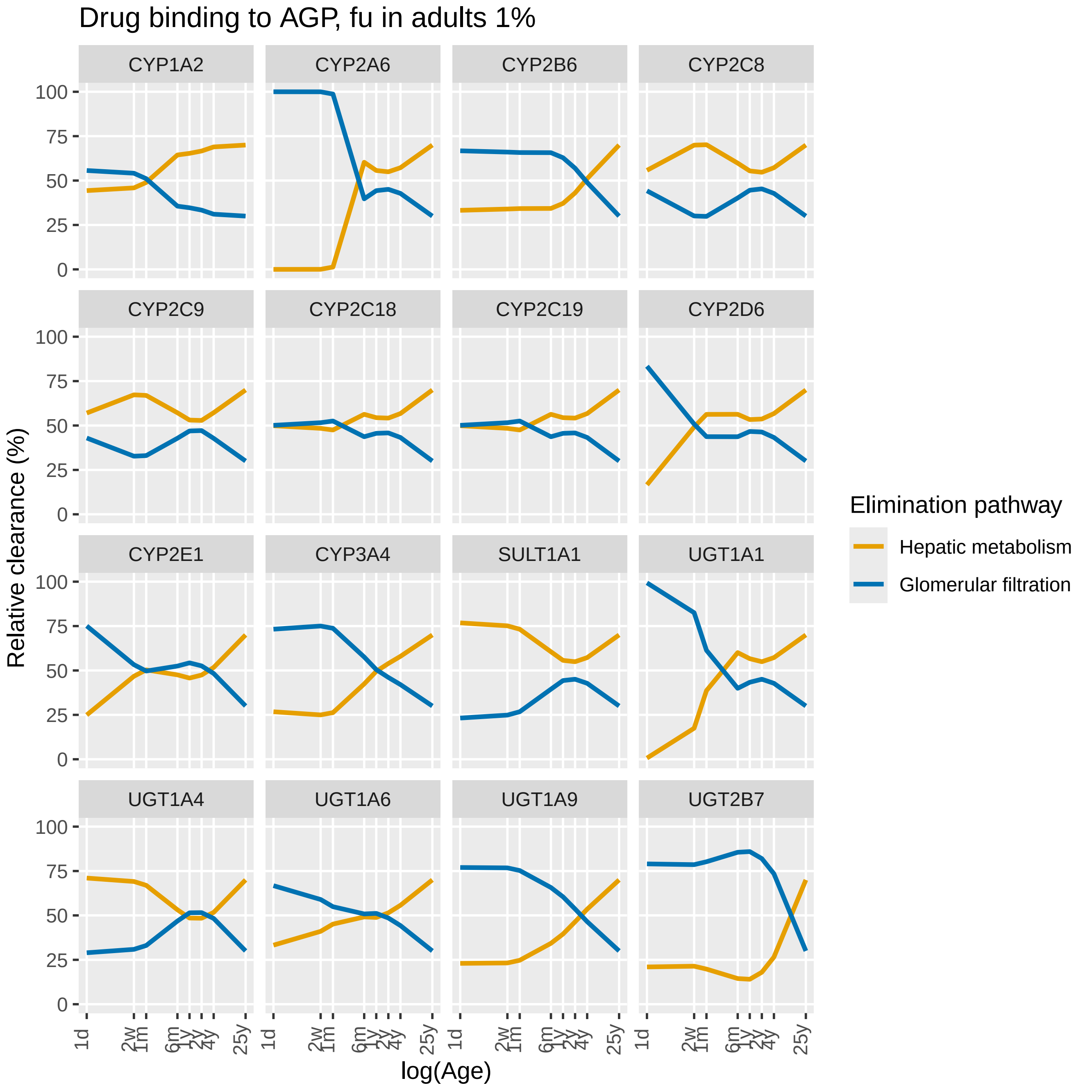

Drug binding to AGP, fu in adults 1%
CYP1A2
CYP2A6
CYP2B6
CYP2C8
CYP2C9
CYP2C18
CYP2C19
CYP2D6
CYP2E1
CYP3A4
SULT1A1
UGT1A1
UGT1A4
UGT1A6
UGT1A9
UGT2B7
100
75
50
25
0
Relative clearance (%)
1d
2w
1m
6m
1y
2y
4y
25y
log(Age)
Elimination pathway
Hepatic metabolism
Glomerular filtration

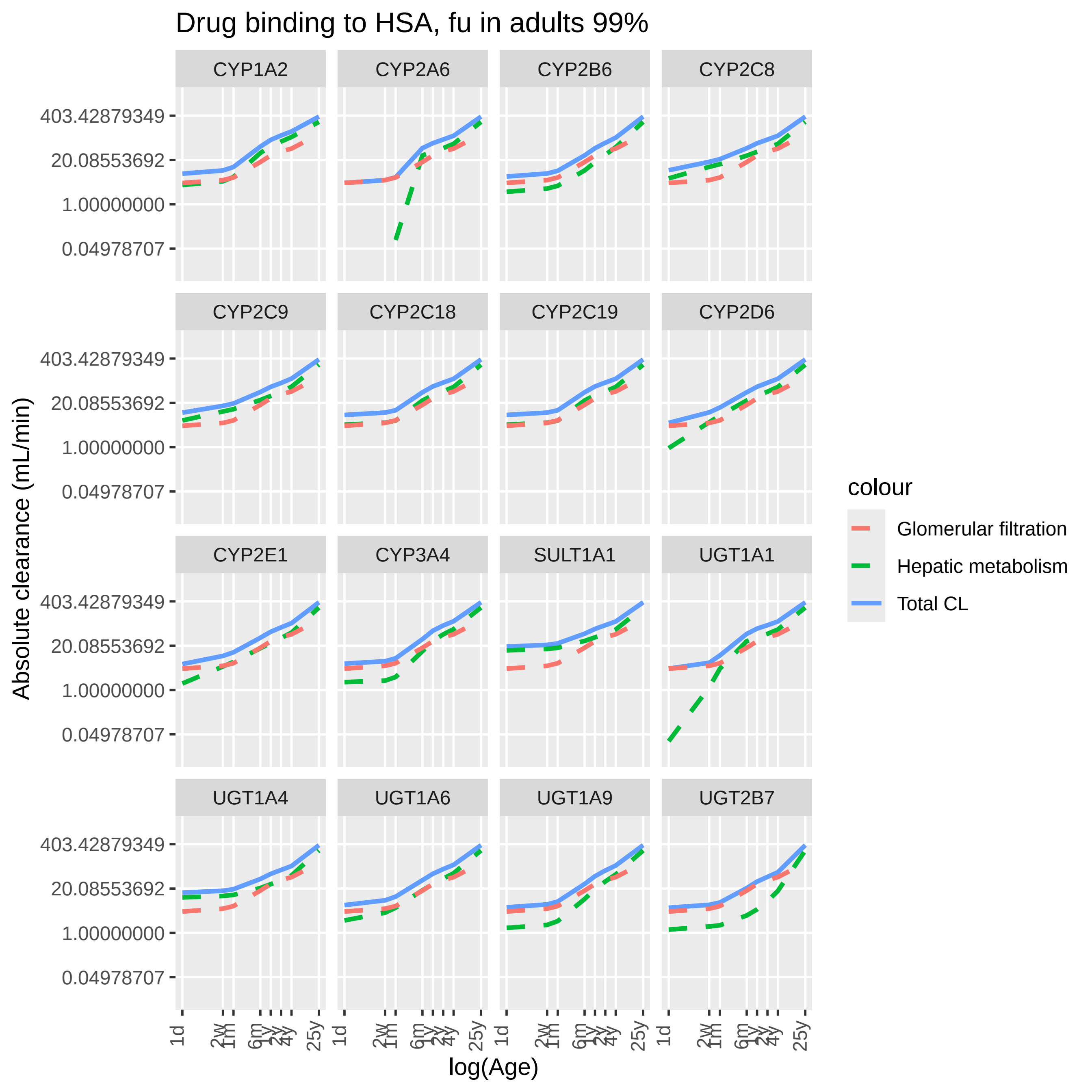

Drug binding to HSA, fu in adults 99%
CYP1A2
CYP2A6
CYP2B6
CYP2C8
CYP2C9
CYP2C18
CYP2C19
CYP2D6
CYP2E1
CYP3A4
SULT1A1
UGT1A1
UGT1A4
UGT1A6
UGT1A9
UGT2B7
403.42879349
20.08553692
1.00000000
0.04978707
Absolute clearance (mL/min)
1d
2w
1m
6m
1y
2y
4y
25y
log(Age)
colour
Glomerular filtration
Hepatic metabolism
Total CL

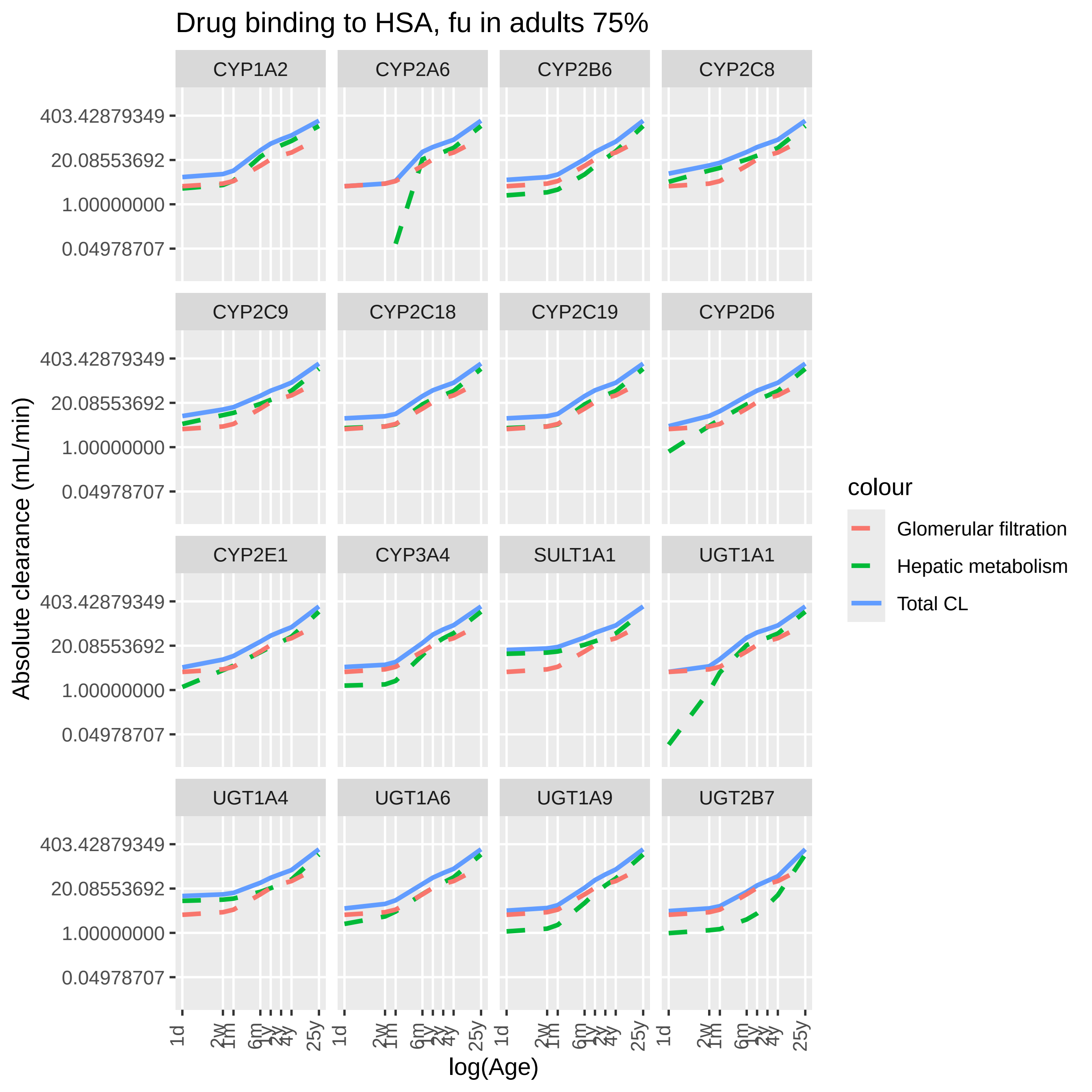
Drug binding to HSA, fu in adults 75%
CYP1A2
CYP2A6
CYP2B6
CYP2C8
CYP2C9
CYP2C18
CYP2C19
CYP2D6
CYP2E1
CYP3A4
SULT1A1
UGT1A1
UGT1A4
UGT1A6
UGT1A9
UGT2B7
403.42879349
20.08553692
1.00000000
0.04978707
Absolute clearance (mL/min)
1d
2w
1m
6m
1y
2y
4y
25y
log(Age)
colour
Glomerular filtration
Hepatic metabolism
Total CL

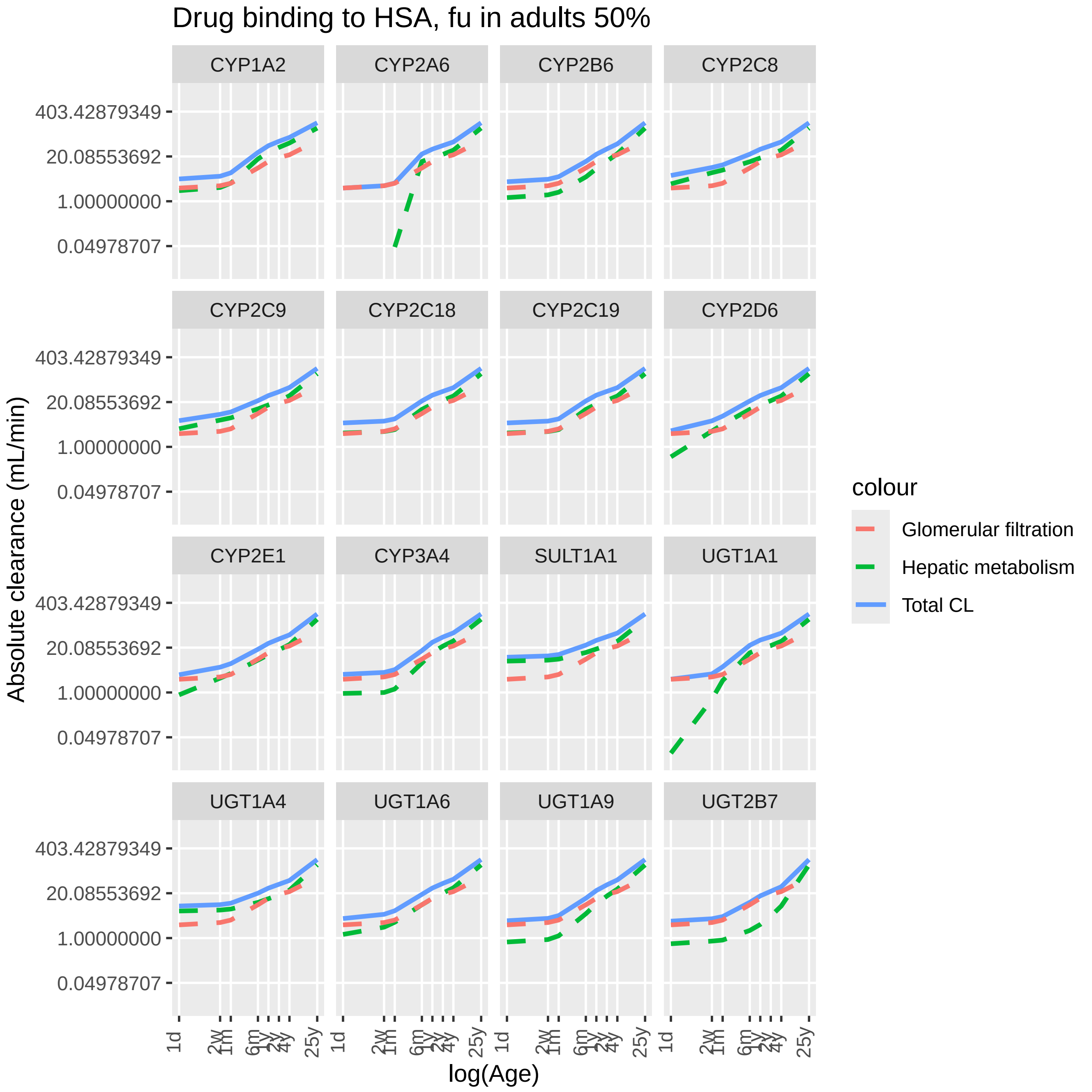

Drug binding to HSA, fu in adults 50%
CYP1A2
CYP2A6
CYP2B6
CYP2C8
CYP2C9
CYP2C18
CYP2C19
CYP2D6
CYP2E1
CYP3A4
SULT1A1
UGT1A1
UGT1A4
UGT1A6
UGT1A9
UGT2B7
403.42879349
20.08553692
1.00000000
0.04978707
Absolute clearance (mL/min)
1d
2w
1m
6m
1y
2y
4y
25y
log(Age)
colour
Glomerular filtration
Hepatic metabolism
Total CL

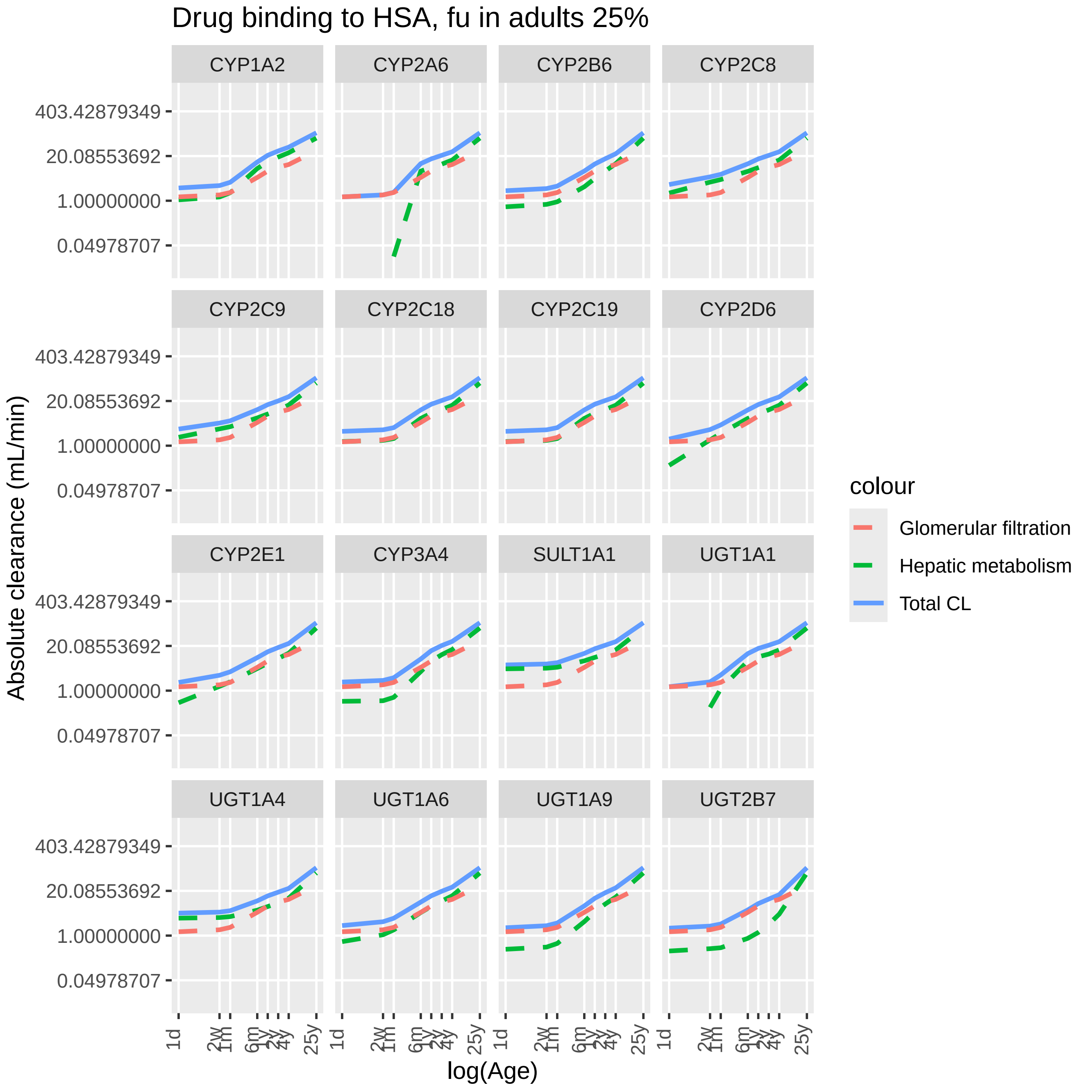

Drug binding to HSA, fu in adults 25%
CYP1A2
CYP2A6
CYP2B6
CYP2C8
CYP2C9
CYP2C18
CYP2C19
CYP2D6
CYP2E1
CYP3A4
SULT1A1
UGT1A1
UGT1A4
UGT1A6
UGT1A9
UGT2B7
403.42879349
20.08553692
1.00000000
0.04978707
Absolute clearance (mL/min)
1d
2w
1m
6m
1y
2y
4y
25y
log(Age)
colour
Glomerular filtration
Hepatic metabolism
Total CL

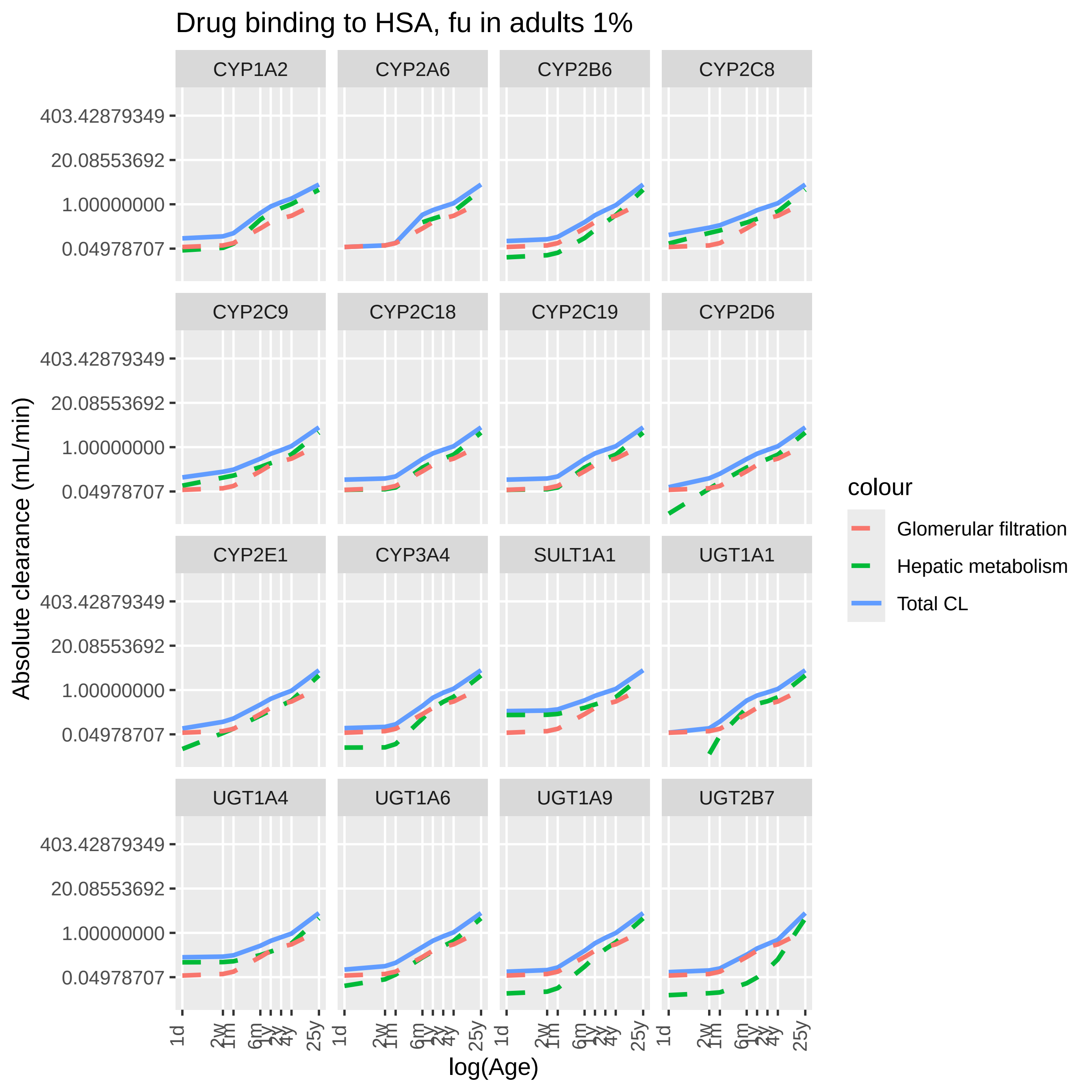
Drug binding to HSA, fu in adults 1%
CYP1A2
CYP2A6
CYP2B6
CYP2C8
CYP2C9
CYP2C18
CYP2C19
CYP2D6
CYP2E1
CYP3A4
SULT1A1
UGT1A1
UGT1A4
UGT1A6
UGT1A9
UGT2B7
403.42879349
20.08553692
1.00000000
0.04978707
Absolute clearance (mL/min)
1d
2w
1m
6m
1y
2y
4y
25y
log(Age)
colour
Glomerular filtration
Hepatic metabolism
Total CL

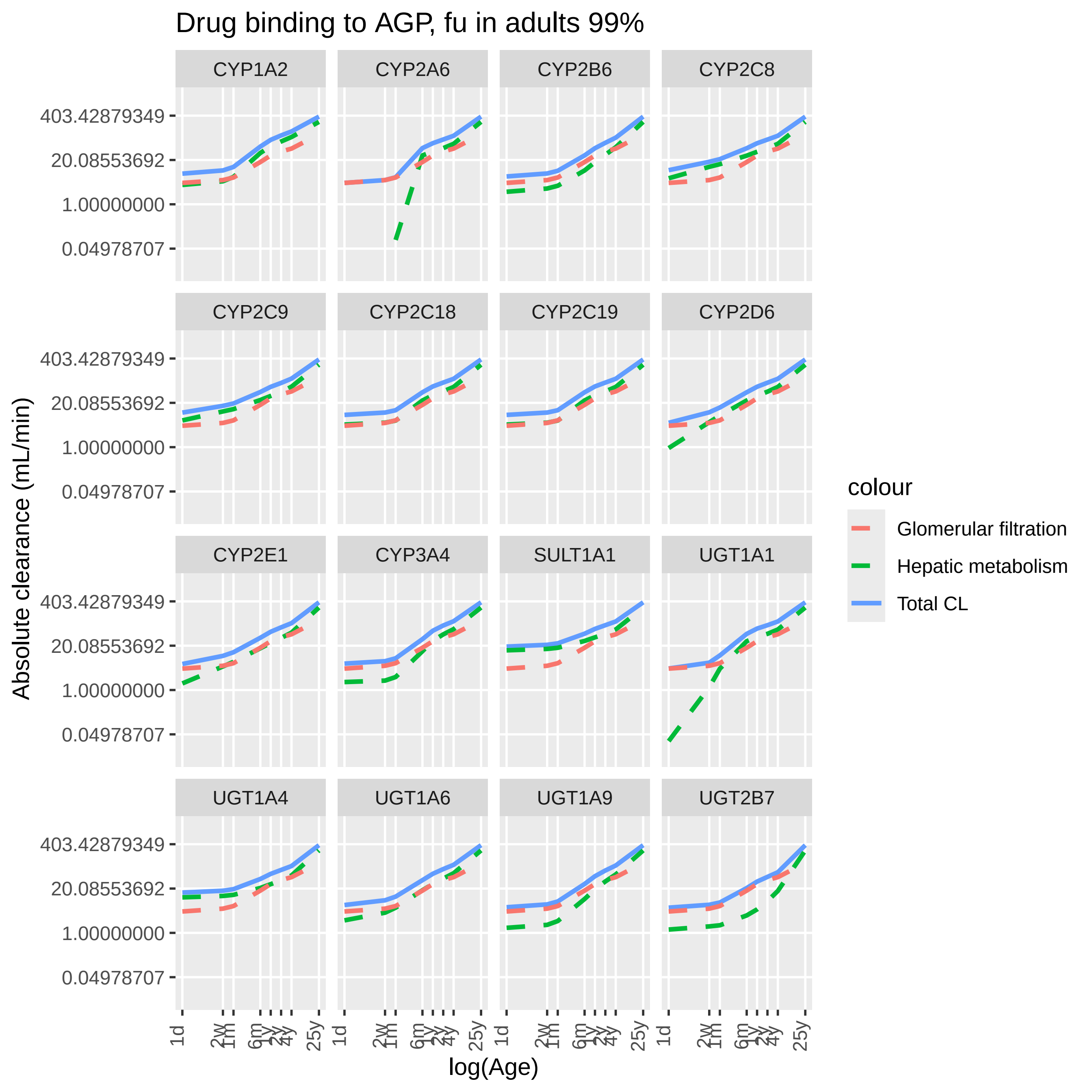

Drug binding to AGP, fu in adults 99%
CYP1A2
CYP2A6
CYP2B6
CYP2C8
CYP2C9
CYP2C18
CYP2C19
CYP2D6
CYP2E1
CYP3A4
SULT1A1
UGT1A1
UGT1A4
UGT1A6
UGT1A9
UGT2B7
403.42879349
20.08553692
1.00000000
0.04978707
Absolute clearance (mL/min)
1d
2w
1m
6m
1y
2y
4y
25y
log(Age)
colour
Glomerular filtration
Hepatic metabolism
Total CL

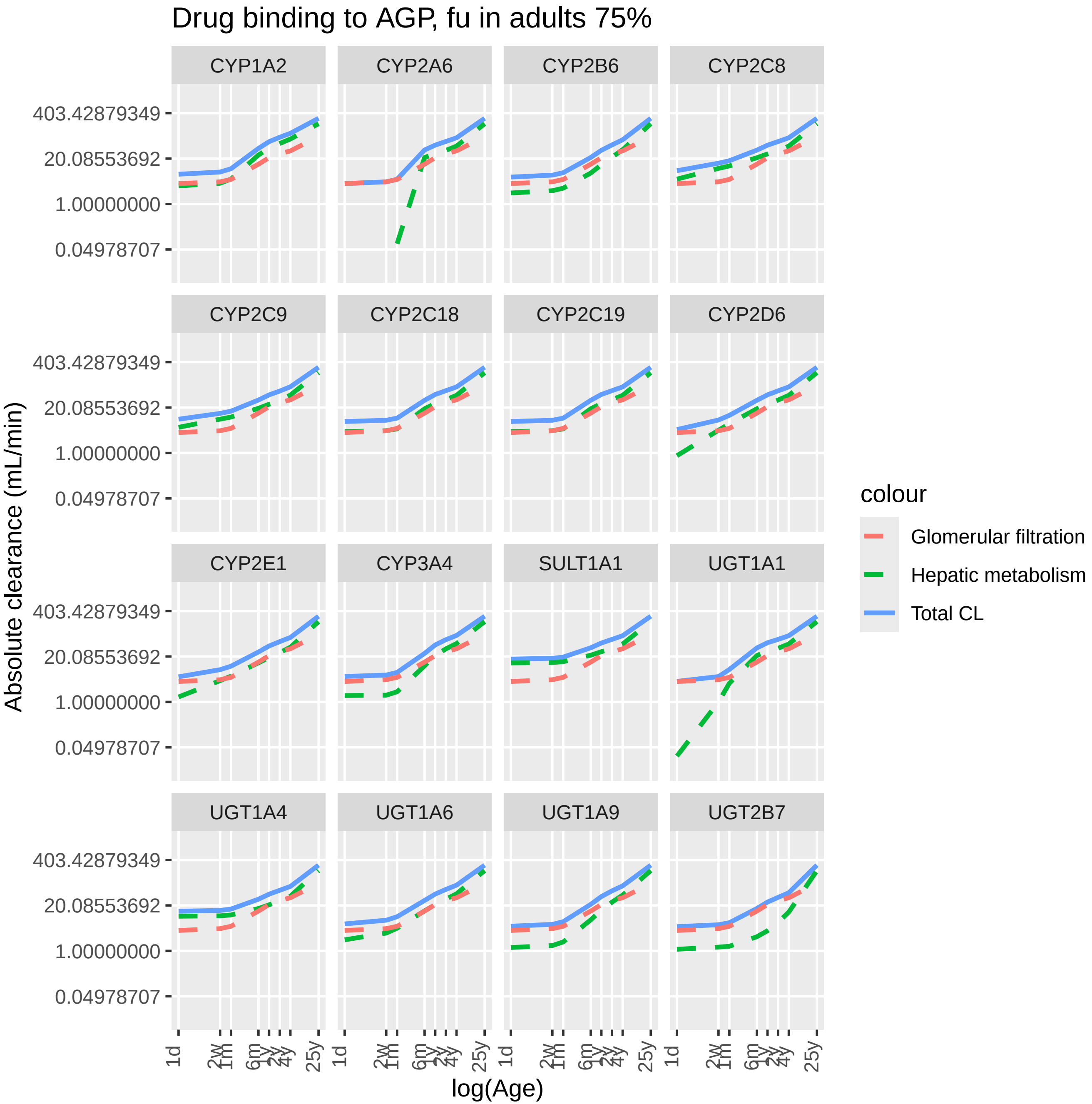
Drug binding to AGP, fu in adults 75%
CYP1A2
CYP2A6
CYP2B6
CYP2C8
CYP2C9
CYP2C18
CYP2C19
CYP2D6
CYP2E1
CYP3A4
SULT1A1
UGT1A1
UGT1A4
UGT1A6
UGT1A9
UGT2B7
403.42879349
20.08553692
1.00000000
0.04978707
Absolute clearance (mL/min)
1d
2w
1m
6m
1y
2y
4y
25y
log(Age)
colour
Glomerular filtration
Hepatic metabolism
Total CL

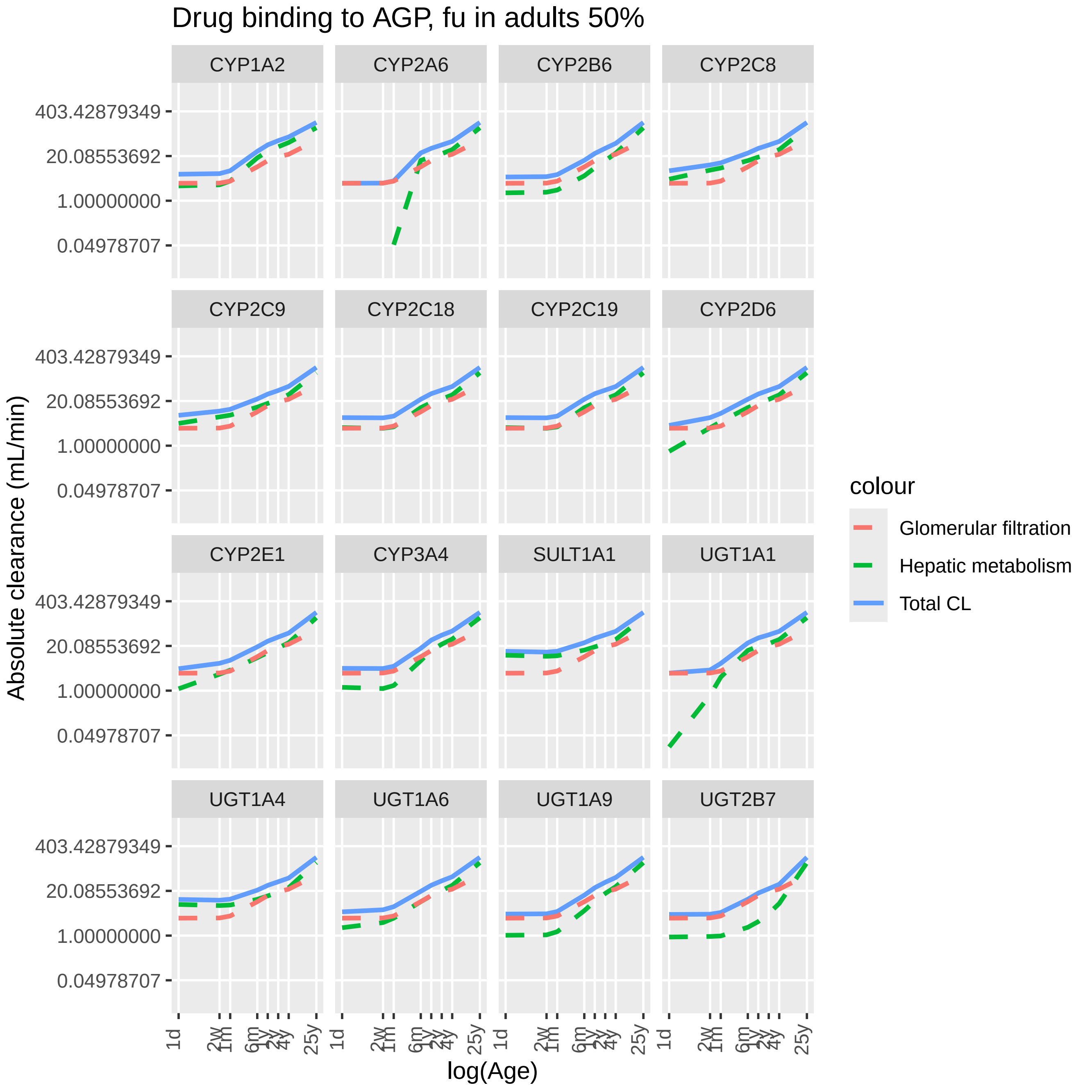

Drug binding to AGP, fu in adults 50%
CYP1A2
CYP2A6
CYP2B6
CYP2C8
CYP2C9
CYP2C18
CYP2C19
CYP2D6
CYP2E1
CYP3A4
SULT1A1
UGT1A1
UGT1A4
UGT1A6
UGT1A9
UGT2B7
403.42879349
20.08553692
1.00000000
0.04978707
Absolute clearance (mL/min)
1d
2w
1m
6m
1y
2y
4y
25y
log(Age)
colour
Glomerular filtration
Hepatic metabolism
Total CL

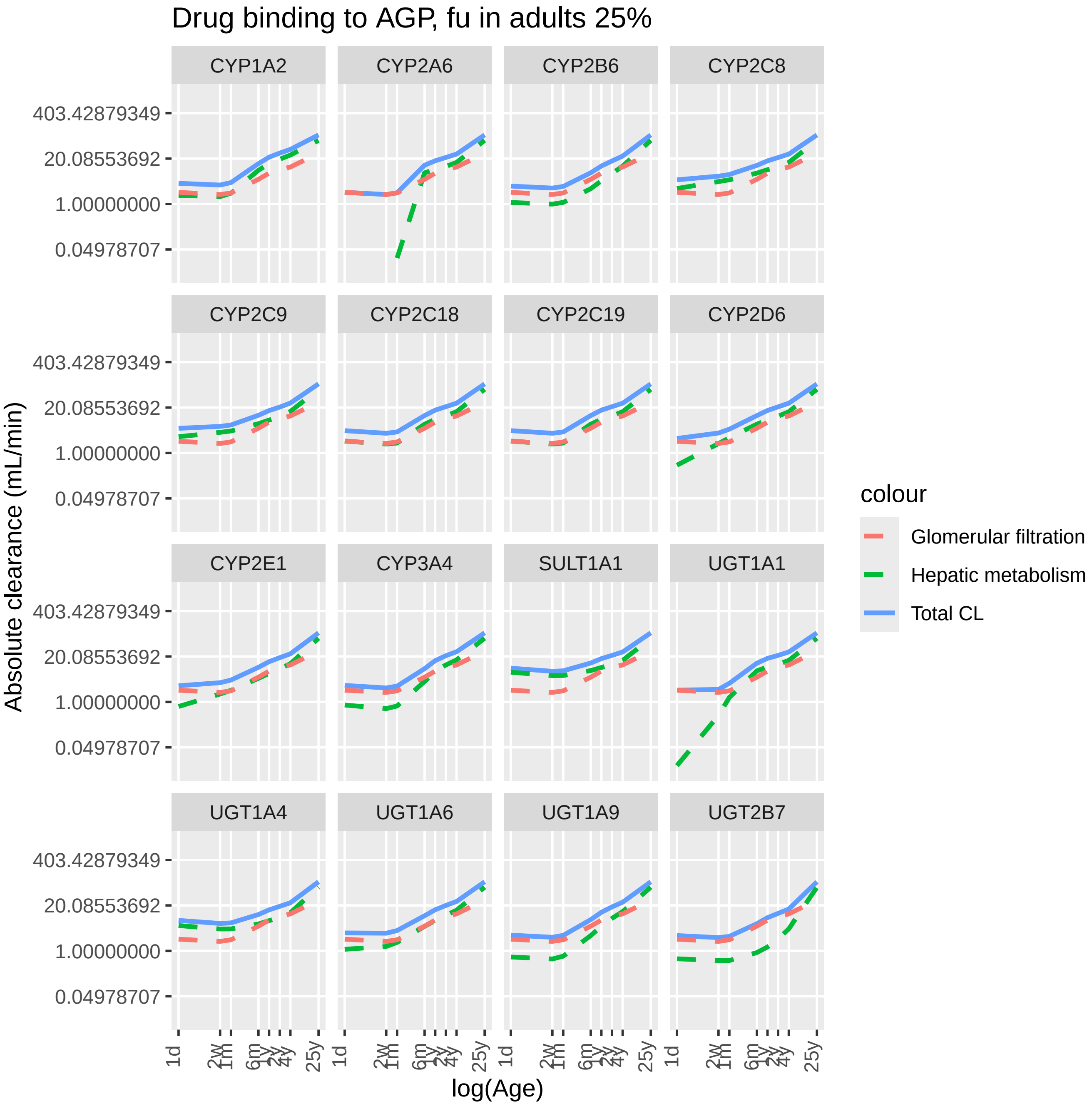

Drug binding to AGP, fu in adults 25%
CYP1A2
CYP2A6
CYP2B6
CYP2C8
CYP2C9
CYP2C18
CYP2C19
CYP2D6
CYP2E1
CYP3A4
SULT1A1
UGT1A1
UGT1A4
UGT1A6
UGT1A9
UGT2B7
403.42879349
20.08553692
1.00000000
0.04978707
Absolute clearance (mL/min)
1d
2w
1m
6m
1y
2y
4y
25y
log(Age)
colour
Glomerular filtration
Hepatic metabolism
Total CL

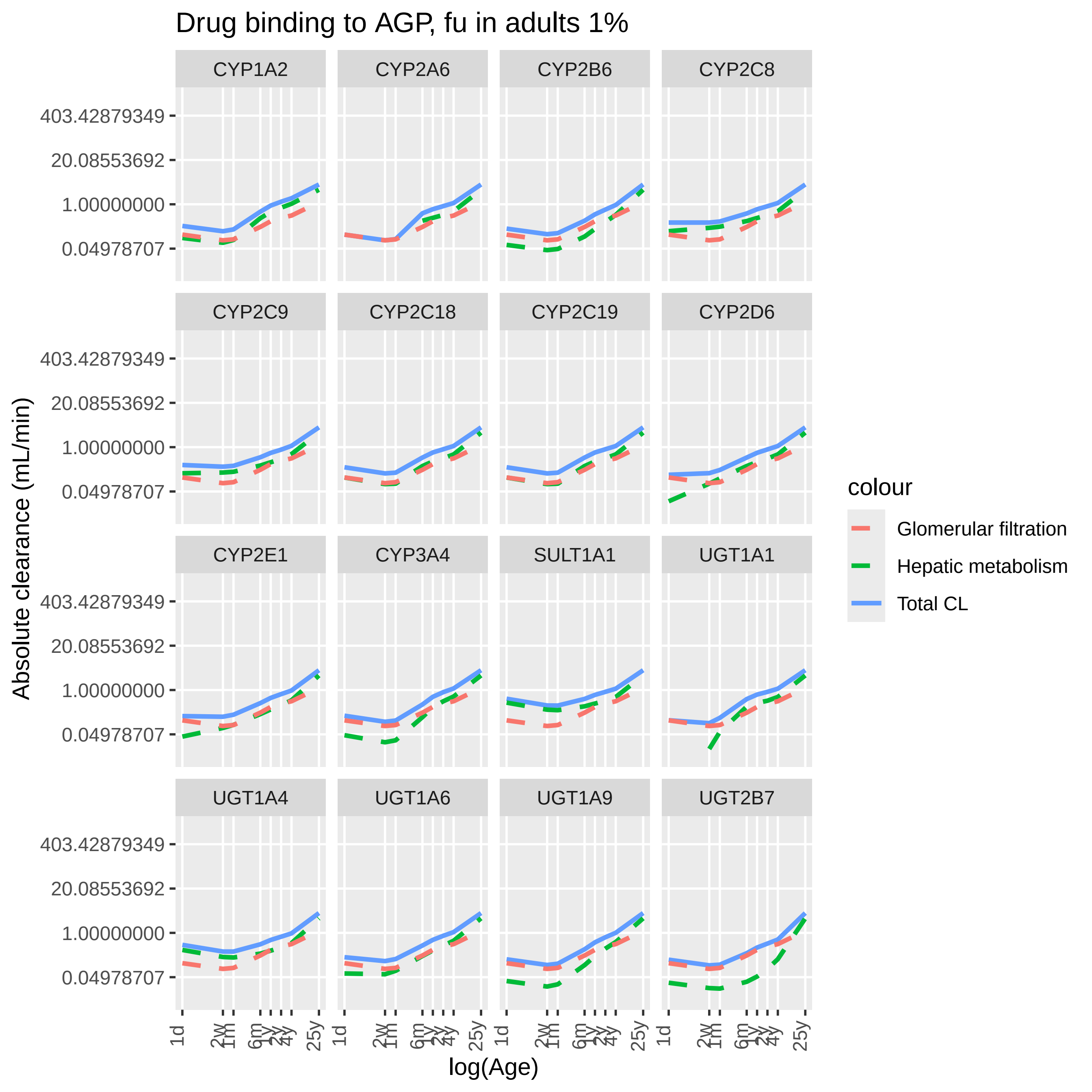
Drug binding to AGP, fu in adults 1%
CYP1A2
CYP2A6
CYP2B6
CYP2C8
CYP2C9
CYP2C18
CYP2C19
CYP2D6
CYP2E1
CYP3A4
SULT1A1
UGT1A1
UGT1A4
UGT1A6
UGT1A9
UGT2B7
403.42879349
20.08553692
1.00000000
0.04978707
Absolute clearance (mL/min)
1d
2w
1m
6m
1y
2y
4y
25y
log(Age)
colour
Glomerular filtration
Hepatic metabolism
Total CL
